# Identifying Ancient Settlement Patterns through LiDAR in the Mosquitia Region of Honduras

**DOI:** 10.1371/journal.pone.0159890

**Published:** 2016-08-25

**Authors:** Christopher T. Fisher, Juan Carlos Fernández-Diaz, Anna S. Cohen, Oscar Neil Cruz, Alicia M. Gonzáles, Stephen J. Leisz, Florencia Pezzutti, Ramesh Shrestha, William Carter

**Affiliations:** 1 Department of Anthropology, Colorado State University, Fort Collins, Colorado, United States of America; 2 National Center for Airborne Laser Mapping, University of Houston, Houston, Texas, United States of America; 3 Department of Anthropology, University of Washington, Seattle, Washington, United States of America; 4 Instituto Hondureño de Antropología e Historia, Tegucigalpa, Honduras; 5 Independent Scholar, Los Angeles, California, United States of America; Max Planck Institute for the Science of Human History, GERMANY

## Abstract

The Mosquitia ecosystem of Honduras occupies the fulcrum between the American continents and as such constitutes a critical region for understanding past patterns of socio-political development and interaction. Heavy vegetation, rugged topography, and remoteness have limited scientific investigation. This paper presents prehistoric patterns of settlement and landuse for a critical valley within the Mosquitia derived from airborne LiDAR scanning and field investigation. We show that (*i*) though today the valley is a wilderness it was densely inhabited in the past; (*ii*) that this population was organized into a three-tiered system composed of 19 settlements dominated by a city; and, (iii) that this occupation was embedded within a human engineered landscape. We also add to a growing body of literature that demonstrates the utility of LiDAR as means for rapid cultural assessments in undocumented regions for analysis and conservation. Our ultimate hope is for our work to promote protections to safeguard the unique and critically endangered Mosquitia ecosystem and other similar areas in need of preservation.

## Introduction

**Fig 1:**
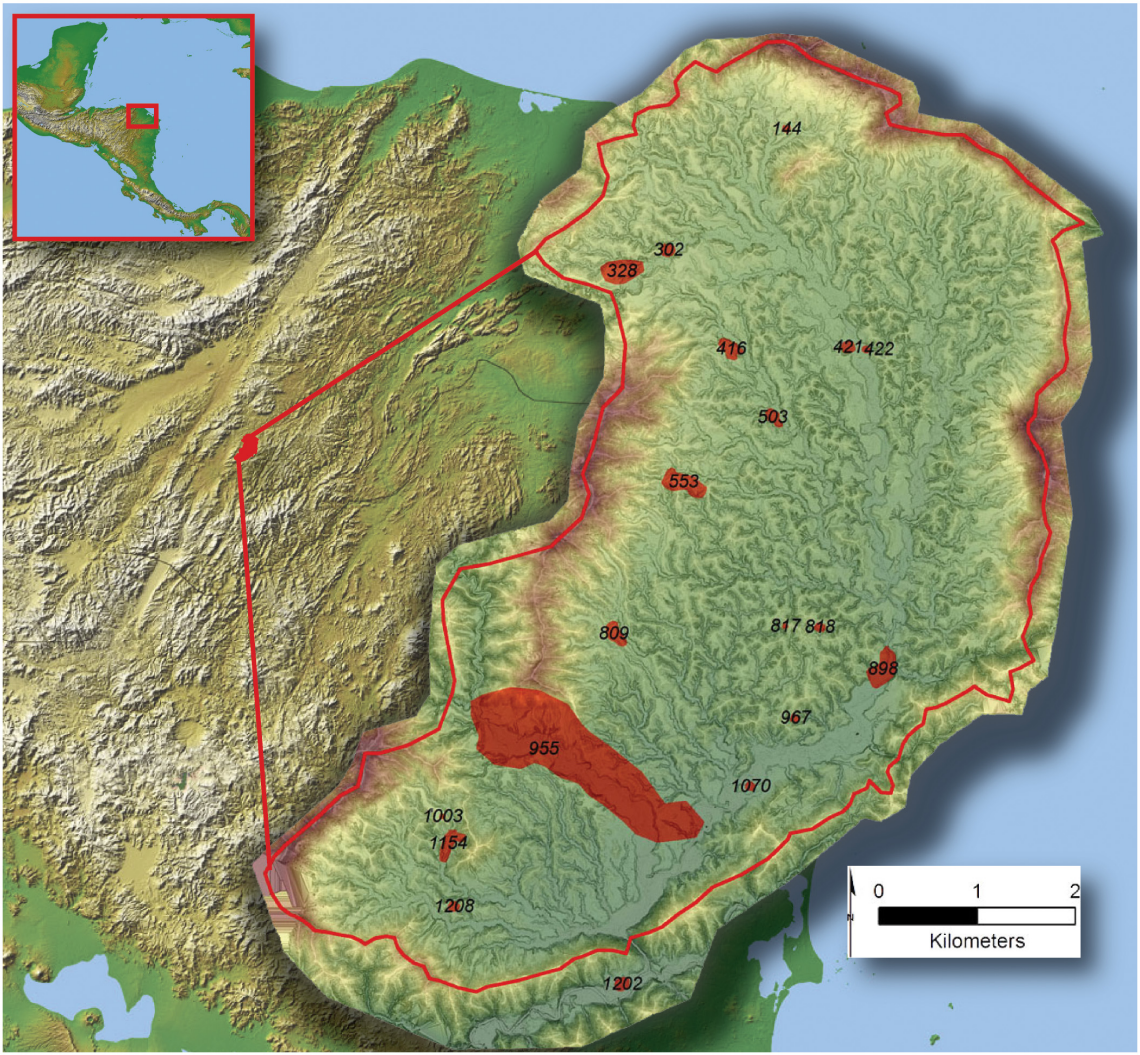
The location of the Valle de la Fortaleza within Central America (inset) and the distribution of prehistoric settlements within the valley superimposed over the Mosquitia region. Settlement extent is shown in red along with an identifier. Settlement numbers were assigned based on a 250 m. grid (fishnet) laid across the area of LiDAR coverage. Solid red line denotes the Valley watershed. All data shown superimposed over the sum of a 16 angle composite hillshade draped on a color shaded NASA SRTM DSM with a resolution of 1 m/pixel.

There is increasing evidence that tropical forest ecosystems in the Americas resulted from long-term trajectories of coupled human-environment systems involving urbanism and landscape engineering though in most areas the scale, timing, and intensity of ancient human impacts remains unknown [1–6]. Such an understanding is critical for a better understanding of diachronic socionatural legacies [7,8] to address questions of broad social importance such as better defining the Anthropocene [9–14] and to create baseline data for climate change science [15]. In archaeology one important element toward understanding ancient urbanism are data detailing the distribution and function of settlements across a region [16–28] and how those settlements relate to the broader landscape [29–32]. Ancient patterns of settlement and land use in tropical regions are critical lacunae to understanding past settlement patterning because such data are challenging to acquire in dense vegetation cover. Here we report on unique research that used field verified LiDAR data to inventory the distribution of settlements in a remote river valley located near the Río Plátano Biosphere Reserve of Honduras (Fig 1) [33–36]. Though some of the earliest archaeology in the Americas comes from this area [37,38], little sustained archaeological research has been undertaken because the heavy vegetation cover, remoteness, and risk of tropical diseases makes traditional techniques impractical. Thus critical questions remain concerning the organization, intensity, and extent of this ancient occupation. How densely settled was the region prior to European contact? What was the spatial nature of these settlements? How did this occupation impact the Mosquitia Environment?

We show that our study region, designated here as the *Valle de la Fortaleza* [*Fortaleza*], was occupied by a network of 19 settlements organized in a three-tiered hierarchy, including one that we characterize as a city which we call *la Ciudad del Jaguar* [*Jaguar*]. This means that though today the valley is a tropical wilderness, in the past it was a densely settled region. This population was supported by an engineered landscape designed to maximize food production and stabilize the environment through a network of terraces and water control features. We also show that the size-distribution of valley settlements is suggestive of a pattern whereby a primary site comes to dominate a region as a result of external socio-political pressure. These include several possibilities such as those related to synoikism, Maya migrants from the Classic period ‘collapse’ [39–43], or the influence of potential paramount chiefdoms like those mentioned by Spanish chroniclers in the sixteenth century [44–47].

The Mosquitia extends across Honduras and Nicaragua and is often called Central America’s little Amazon because the multilevel rainforest and associated ecosystem mimics that of South America and the region is important for assessing the impacts of global climate [48,49]. The region is considered important global patrimony and contains unique and endangered species along with national and international protections, including a UNESCO World Heritage and biosphere designation (Río Plátano Biosphere Reserve), with the region currently classified as threatened [50,51].

Our study region encompasses the Valle de la Fortaleza, a small (26 km^2^) basin near the Río Plátano biosphere (Fig 1). No toponyms are known for this area from maps, indigenous accounts, official records, or the published literature. Forteleza constitutes one of the drainages that form the headwaters of the Río Pao and is surrounded by a large ring of mountains making it naturally fortified. Two unnamed drainages dissect the valley along the cardinal directions and can be forded but are not navigable. The valley contains thin tropical soils in upland areas that give way to alluvial systems on the valley bottom [52,53].

Vegetation is a mix of tropical riparian areas [54] with a mature multi-level rain forest on the uplands [50,55]. The valley supports large populations of fauna including various monkeys (*Cebidae alouatta*; *Cebidae ateles)*, jaguars (*Panthera onca*), peccary (*Tayassu pecari*), and endangered birds (*Ara macao*; *Amazilia luciae*; *Procnias tricarunculatus*) [56–58]. Today the valley is free from roads, farms, and deforestation, but it is threatened by human encroachment and related deforestation less than 20 km away.

Historically NE Honduras is defined as a culturally diverse area between Trujillo and the Río Plátano, including the Bay Islands [59–65]. While eastern parts of the Mosquitia—including our study area—have seen limited scientific research, a rough chronology can be constructed from adjacent work. Recent outlines of previous archaeological work in NE Honduras along with a broad regional summary can be found in Begley [66]) and Cuddy [45]. The earliest occupation occurred during the Cuyamel (1200 BC–AD 600-Period IVb), but the first definitive occupation in the area was during the Selín phase (600 BC–AD 1000-Period IVb), which is thought to mark the advent of social complexity with large planned sites, ballcourts, and a Mosquitia plaza tradition [45]. The Transitional Selín marks the end of the Mesoamerican Classic period and the collapse of adjacent Maya political and economic systems. Regional alliances seem to follow a broader pattern linked to coastal trade with cultural connections to the Gran Nicoya of Costa Rica and the coast, and the adoption of external symbols of power, such as the large grinding stones or seats (metates). The Cocal phase (AD 1000 –AD 1500+) spans the Mesoamerican Postclassic and is represented by the sites of Wankibilia, Las Crucitas de Aner, and Río Claro [47,67]. During the latter portion of this phase, parts of the region may have been dominated by the historically documented Taguzgalpa polity [44,61,63].

## Materials and Methods

Remote sensing techniques have become increasingly common [68–70], especially in areas that are impossible to survey using traditional ‘boots on the ground’ techniques [26]. High resolution airborne mapping LiDAR has recently been applied to archaeological remains in the temperate and neotropical zones of the Americas with dramatic results [33,71–80], though the resulting data is more commonly used for much broader studies [81–85]. Indeed Chase et al. [69] have even termed the application of LiDAR to Mesoamerican archaeology a ‘paradigm shift’.

Through LiDAR scanning a grid of infrared beams is painted onto a landscape and the returns calculated to create a 3 dimensional matrix. The resulting point cloud can then be filtered using computer software to ‘turn off’ sections of the data to highlight features of interest–which may be the ground surface, different levels of vegetation, or other features. These filtered results are most often turned into other 2D+ and 3D products and visualizations such as DEM/DTM/DSM’s, contour maps, hillshades, and other analytic datasets [74]. The resolution of these products is variable and depends on the vegetation, number of passes, quality of the instrument, and many other factors. Importantly these point clouds do not degrade like a photograph but instead persist as a digital database that can be analyzed in the future with increasingly sophisticated analytic tools.

Datasets created from LiDAR are not synonymous with archaeological data created during full coverage survey because point cloud-based data reveal a landscape totality rather than a sampled universe. And full-coverage survey data are sampled in that not all of the human generated features on a landscape can be feasibly recorded. This means that the investigators have made decisions concerning exactly what and how anthropogenic features should be recorded. In contrast LiDAR reveals the totality of the landscape including architecture but also environmental engineering such as terraces, canals, fields, roads, and other human generated imprints. Of course LiDAR must be field-verified meaning that this new scanning technology is not a substitute for ‘boots on the ground’.

LiDAR scanning results in large datasets representing millions of points over large areal scales that are just beginning to revolutionize not only the way that data is collected but importantly the way that archaeological sites and landscapes are analyzed and conserved. This means that point cloud data represent a universe of human generated landscape change that can include sites but also the spaces between [86]. In this sense the advent of ‘big data’ to the study of the past means that archaeology has finally entered its own ‘age of discovery’. It also allows the conservation of archaeological resources in unique ways that can potentially revolutionize our understanding of the past.

**Fig 2:**
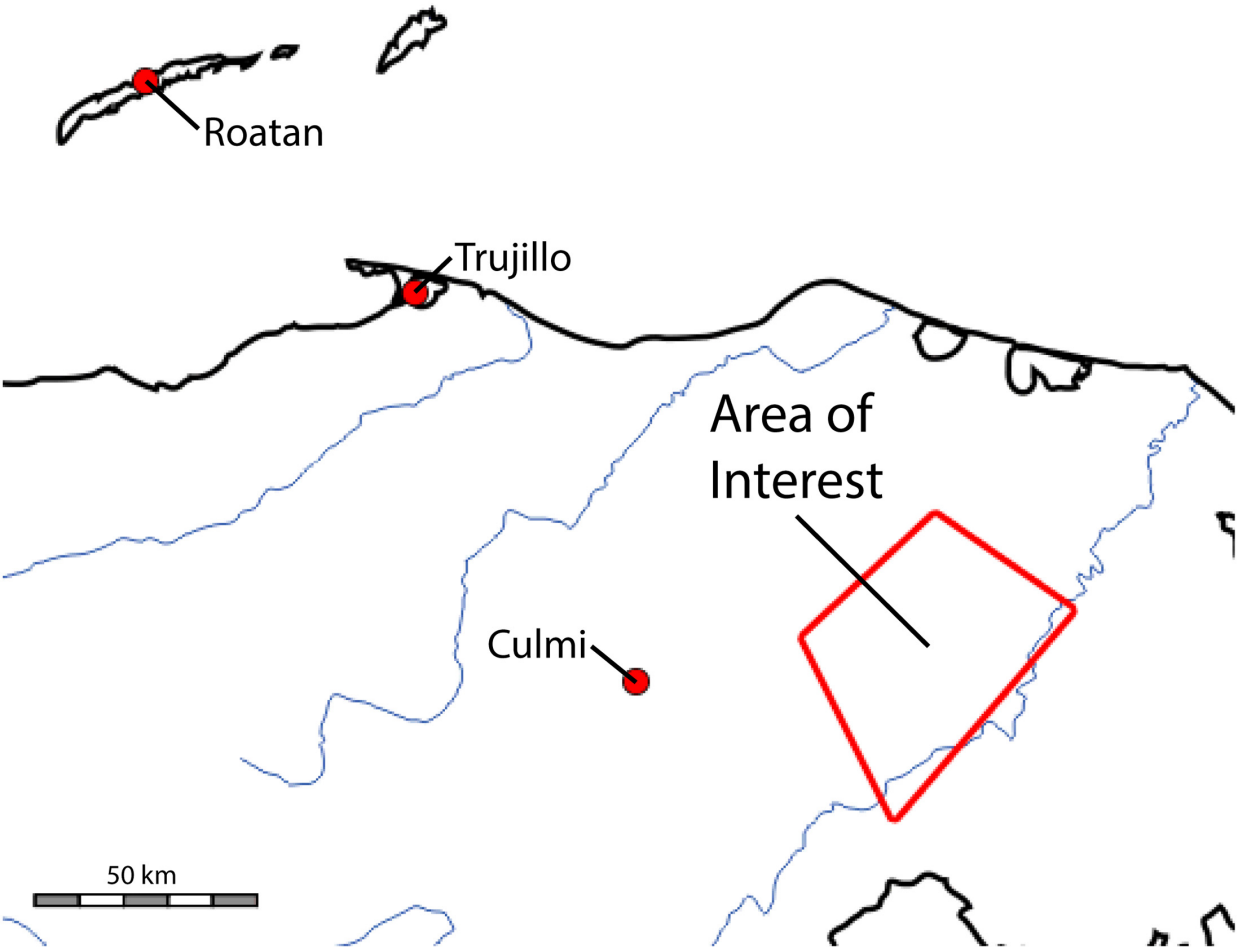
Map of the greater Mosquitia Biosphere showing the LiDAR area of interest and GPS reference stations.

The remoteness of the study region presented unique technical and logistical challenges that were overcome using airborne mapping LiDAR technology, which provides the highest resolution topographical data possible including under thick forest canopy, and has been proven effective to map ancient settlements [87]. All necessary permits were obtained for the described study, which complied with relevant regulations, including those of the Instituto Hondureño de Antropología e Historia (IHAH). Permits for this work were secured by the IHAH in accordance with Honduran law. Secure locations were identified to install and maintain continually operating Global Positioning System (GPS) base stations within 100 kilometers of the project mapping areas and along the trajectory of the aircraft. These baseline distances were critical for compiling precise aircraft trajectories for LiDAR data accuracy. As shown in Fig 2, three GPS base stations were deployed starting at the operational base near the airport in Roatán (Bay Islands), a second near the ocean/land interface near Trujillo (Colón), and a third near Dulce Nombre de Culmi (Olancho) close to the study areas. This allowed for precise aircraft trajectory determination along the entire flight path.

**Fig 3:**
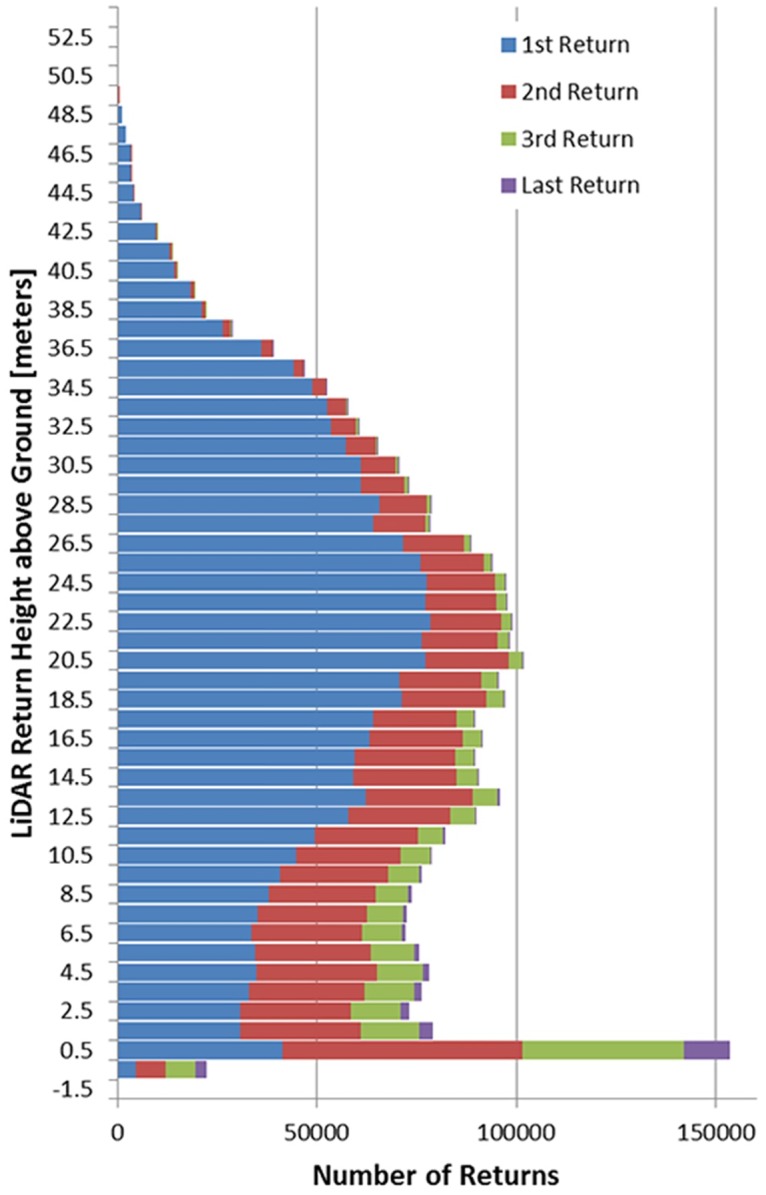
Histogram of forest canopy returns from a 250 m x 250 m sample. The histogram shows the relative occurrence of canopy returns with respect to their height above the modeled ground (DTM). It also shows for each height-above-ground bin the relative distribution of first, second, third, and last returns of each laser pulse.

To ensure to proper representation of the ground surface underneath the forest canopy that allows for an unambiguous identification of potential cultural elevation anomalies a customized flight plan and system configuration were designed. These plans and configurations are aimed at maximizing the sampling density (laser pulse repetition frequency) while maintaining sufficient pulse energy so that the laser energy can propagate on it two-way path through the thick tropical canopy. Fig 3 presents a histogram of LiDAR return heights above the local ground synthesized from all returns from a 250 m x 250 m square located near one of the identified archaeological sites. The histogram is a representation of canopy structure and density for the areas mapped, and shows that the canopy of the rainforest typically consists of multiple levels. The tallest trees extend to a maximum height of 52 m above the ground while the maximum canopy density is reached at about 25 m above the ground. There is also a significant lower, very dense layer of vegetation from 10 m down to about 2 m above ground. For the particular area used to generate Fig 3, only 1.5% of all the detected returns were classified as corresponding to the ground. This low percentage of ground returns as compared to the total number of returns indicate the high closure and density of the tropical canopy and illustrates the need for careful designed flight plan and system configuration to obtain the highest number of ground returns possible.

A previous exploratory project employed spaceborne Synthetic Aperture Radar (SAR) to penetrate the canopy in the Mosquitia region, obtaining some degree of success [88]. The results obtained were relatively low-resolution pixilated images that did not provide unambiguous indications of archaeological features. To maximize the possibility of obtaining ground returns through the thick canopy and to provide unambiguous images, the LiDAR sensor was configured and flight plans were designed to scan every square meter of the rainforest from four different angles, achieving multi-pass full surface illumination.

The flight plans were based on nominal flying heights of 600 m above ground level (AGL) and a ground speed of 60 m/sec. The LiDAR unit was configured so that the laser fired at 125 kHz with a divergence of 0.8 mrad (0.48 m footprint diameter). Higher laser pulse repetition frequencies (PRF) were also tested in an attempt to achieve higher shot densities, but the reduction in energy per pulse at the higher laser pulse rates, resulted in fewer detectable returns from the ground surface. The scanning was performed at ±15° and 60 Hz. The final operating parameters resulted in a minimum shot density of 25 shots/m², with multiple detected returns per laser shot

Based on the system configuration and the available time and budget, it was decided to map three remote river valleys within a 3000 km² section of the Mosquitia region, shown as a red polygon in Fig 2. Only one of these (T1 –the V*alle de la Fortaleza*) is discussed here. Great care was taken to select potential scanning zones so that a complete drainage system was included. In this sense, though we did not know if archaeological remains would be present in the scanned areas, a complete archaeological region was selected. It was our hope that by extension this would enable us to elucidate a complete, internally differentiated human settlement system that could potentially yield much information about cultural systems in the region.

Prior to the LiDAR flights, 60 cm resolution multi-spectral satellite images (Digital Globe) obtained in the visible and near infrared ranges were digitally combined in several band ratios and analyzed using vegetation indices, decorrelation stretching, and anomaly detection. This manipulation highlights areas with spectral anomalies which can be the result of archaeological features affecting the spectral characteristics of the overlying vegetation [89]. The data obtained from this image processing was used to aid in the identification of potential anomalies within the areas of interest and to prioritize the flights. The comparison of the results obtained from the satellite imagery analysis and the LiDAR are beyond the scope of this paper, however it is important to establish that the different methodologies highlight different types of signatures. The imagery analysis might revel spectral anomalies while the lidar revela elevation anomalies, that might or not be spatially or culturally correlated.

Over a two-week period, seven flights were executed totaling 32.1 hours and 8.4 hours of Laser-On-Time, with a total of 3.5 billion laser pulses fired over a combined area of 122.8 km². Of the shots fired, only 2.9 billion were processed (mainly due to cutoff at the edge of the swath to reduce scan edge artifacts), yielding a total of 4.5 billion returns, where only 87 million (1.9% of returns) were classified as ground returns using the algorithm described by Axelsson [90]. From the filtered ground returns, bare-earth digital elevation models (DSMs) were generated from which different types of shaded relief and other products were generated. In addition, contour maps were created from the filtered point cloud data at a number of resolutions. The LiDAR work netted products with a DSM that had a minimum pixel size of 1 meter and a minimum contour interval of 25 cm with 90% confidence. This means that we can identify anomalies on the ground larger than 1 m on a side and over 50 cm in height. Through the ground verification efforts we were able to identify smaller features but given that the average roofed area of a house in Mesoamerica is 62 m2 [91] we feel that the majority of activity areas can be identified. There are however circumstances that can limit this detection; for further discussion see [35].

All of these data were input into an ESRI geodatabase and Informed by our previous survey and LiDAR work in Mexico [87, 92–95], we were able to create a simple archaeological typology and correlating data dictionary. For the Honduras work we used a simplified architectural and topological typology based on general categories that we identified in previous examinations of the LiDAR data. Given the lack of baseline data for the architecture of the region, these characterizations are based mostly on morphology as described in Table 1. It should be noted that an additional category of ‘cultural unknown’ was used for features that look anomalous but did not conform to the typology and that need to be confirmed on the ground in the future.

**Table 1:** Simplified architectural and landscape typology used in this study. In the ESRI Geodatabase each category corresponds to a shapefile which then forms the basis for a data dictionary used in the handheld Trimble GPS units.

Category Title	Description	Number identified
**ActiveStream**	Denotes the active, year-round, stream channel as determined from aerial photos, LiDAR data, and field observation.	
**Canal unk (Canal not possible to field check)**	These are likely a mix of prehistoric canals with some relict stream channels. The location, distribution, and relationship between these features point to a cultural origin. Unfortunately due to the heavy cover and landscape position, we were not able to see these features in the field. Many of these features are positioned in the appropriate place for irrigation. For example, an ideal place for a feeder canal is on the outside of a curve at the edge of the floodplain. The linear patterning that is perpendicular to the stream direction is especially suspicious.	300
**Hydrology**	Denotes arroyos or barrancas that are seasonally active. Many of these seasonal streams post-date site abandonment as they cut across cultural features.	
**Terrace area**	These colored zones represent areas of terraces that are too indistinct or damaged to map individually. They can represent both habitation zones (wider terraces) and agricultural features (narrow terraces).	3 km2
**Cultural unk (cultural feature unknown)**	These features denote areas that are likely cultural in origin but will need to be field-checked in the future.	78
**Erosion**	Usually attached or associated with ‘Hydrology,’ these areas mark zones of significant erosion. Much of the erosion cuts through cultural zones and features meaning that it post-dates site abandonment.	
**Terraces**	Here we denote areas of obvious terracing or in some instances platforms. Most of these features are wide (>2 m) and so are most likely are associated with residential zones or connective areas of the site.	200
**Plaza**	These areas represent large, flat, prepared spaces usually flanked by mounds or other cultural features.	45
**Edificios**	Edificios (buildings) are defined by one or more foundational elements visible on the surface. At la Ciudad del Jaguar these occur as linear arrangements of rock and rubble less than 30 cm in height. Some of these features are visible in LiDAR visualizations but the course ‘sieve’ size of the DSM makes interpretation difficult.	48
**Mounds**	A raised earthen platform minimally 50 cm in height and starting at 1 m in width.	205

To aid in the labeling and identification of archaeological sites, a 250 X 250 m grid (fishnet) was laid over the entire valley and numbered sequentially starting in the southwest corner of the scanned area. All sites and archaeological features were given a unique identifier that started with this grid number. Using a combination of ESRI ArcScene (3d) and ArcGIS (2d+), data from the geodatabase was systematically examined for mounded architecture within each grid square. Based on the architectural typology presented in Table 1, identified features were digitized and clusters were assigned numbers based on the associated grid square. It should be noted that some features do not show up in 2D+ products, like contours, but are readily visible in a true 3D view in ArcScene. All statistics described below were calculated in ArcGis.This analysis was conducted manually by the investigators in this paper. and each area was visually inspected at least three times by each investigator.

Informed by the previous LiDAR and GIS analysis, the 2015 ground-truthing effort used a methodology that combined aspects of full-coverage survey LiDAR scanning and sampling to document sites within the Valley. We used a targeted sampling strategy to confirm the different classes of architectural features identified in the initial LiDAR assay, document major mound groups, and conduct surface collection of artifacts. Using Trimble handheld GPS surveying instruments with real-time sub-meter accuracy, field teams were able to view their location relative to LiDAR derived products (such as Hillshades, DEMs, contour maps etc.), along with annotated shapefiles. Field verification efforts were designed to confirm that cultural features were present and time investment at each site was minimal. The heavy vegetation cover limited our ability to conduct surface collection of artifacts and hindered verification work. We estimate that we were able to sample roughly 40% of the features at the main site of Jaguar. Based on previous LiDAR work in Mexico, we believe that this represents an adequate sample to confirm the cultural validity of the sites and architectural features identified during the GIS phase of the project.

Given the density of vegetation, remoteness of the area, and cost of helicopter time for further survey we were not able to field check any of the smaller occupations identified in the initial assay of the LiDAR data. Deposits such as artifact scatters and those not represented by anthropogenic topographic change cannot be identified using LiDAR data alone. Likewise the dense vegetation of the region also means that cultural material is not visible on the surface and would be missed using traditional methods. Thus, we were able to document zones of human modified landscape coupled with mounds and other features, but not the total spatial extent of each occupation.

Archaeological settlement pattern data are by definition incomplete [96,97] and in this study we were not able to include in this inventory smaller settlements or zones of occupation that would have contained structures of wattle and daub or other perishable materials not visible on the surface. Aside from Jaguar, we have no diachronic data about these sites which, given the dense vegetation cover, would have to come from an intensive program of excavation as little cultural material is visible on the surface. This means practically that the site areas visible through LiDAR are much smaller than the actual areas of occupation. Realistically, given the remoteness of this area, the cost of fieldwork, and archaeological priorities, the sites documented in this inventory will not be systematically investigated or even visited similar to other archaeological regions. LiDAR may be the only record of these locations before they are damaged or destroyed through deforestation and associated modern landuse.

## Results and Discussion

**Fig 4:**
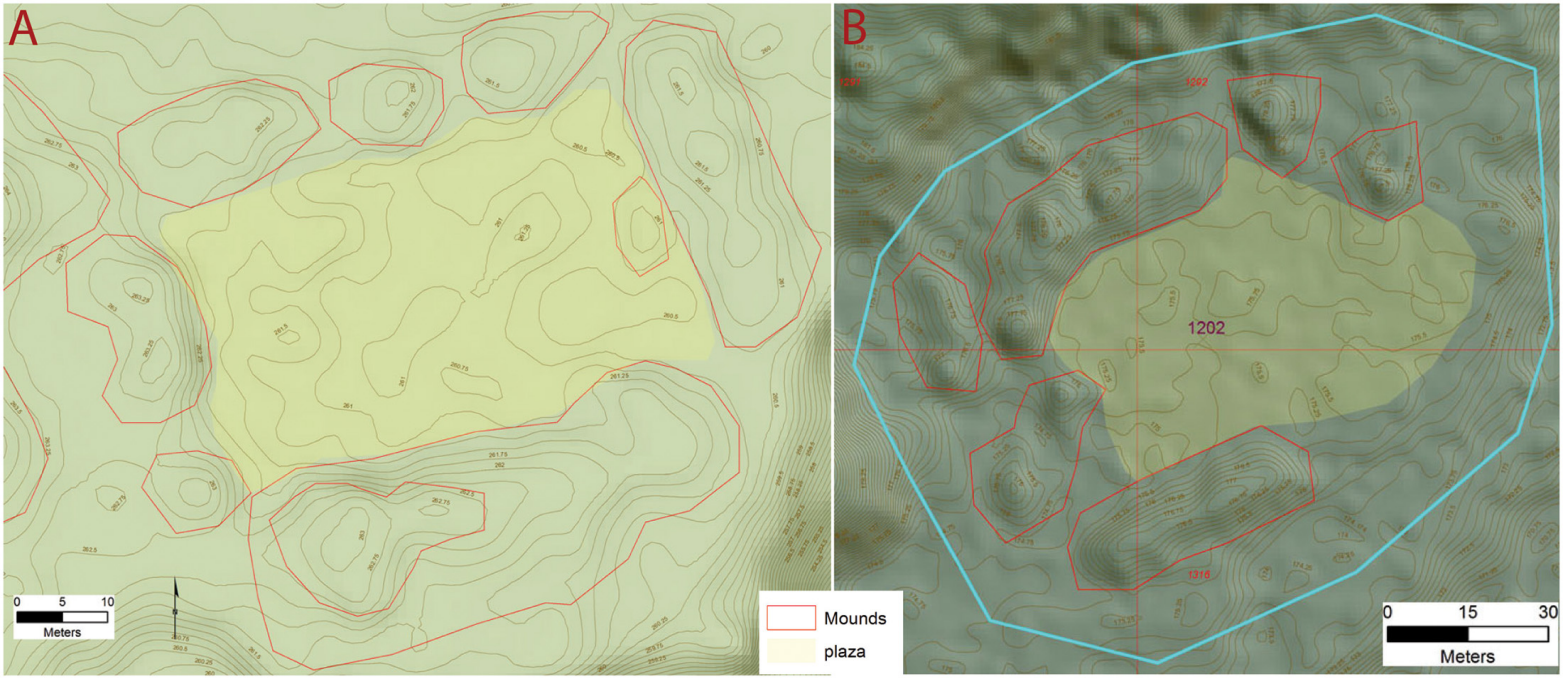
Examples of square plaza configurations (Type A). A) Plaza group from site 955 Jaguar; B) Site 1202. Digitized features are shown over a composite hillshade view taken from 16 different angles draped on a color shaded DSM with a resolution of 1 m/pixel. Contour interval is 25 cm. All visualizations created using high resolution aerial LiDAR.

**Fig 5:**
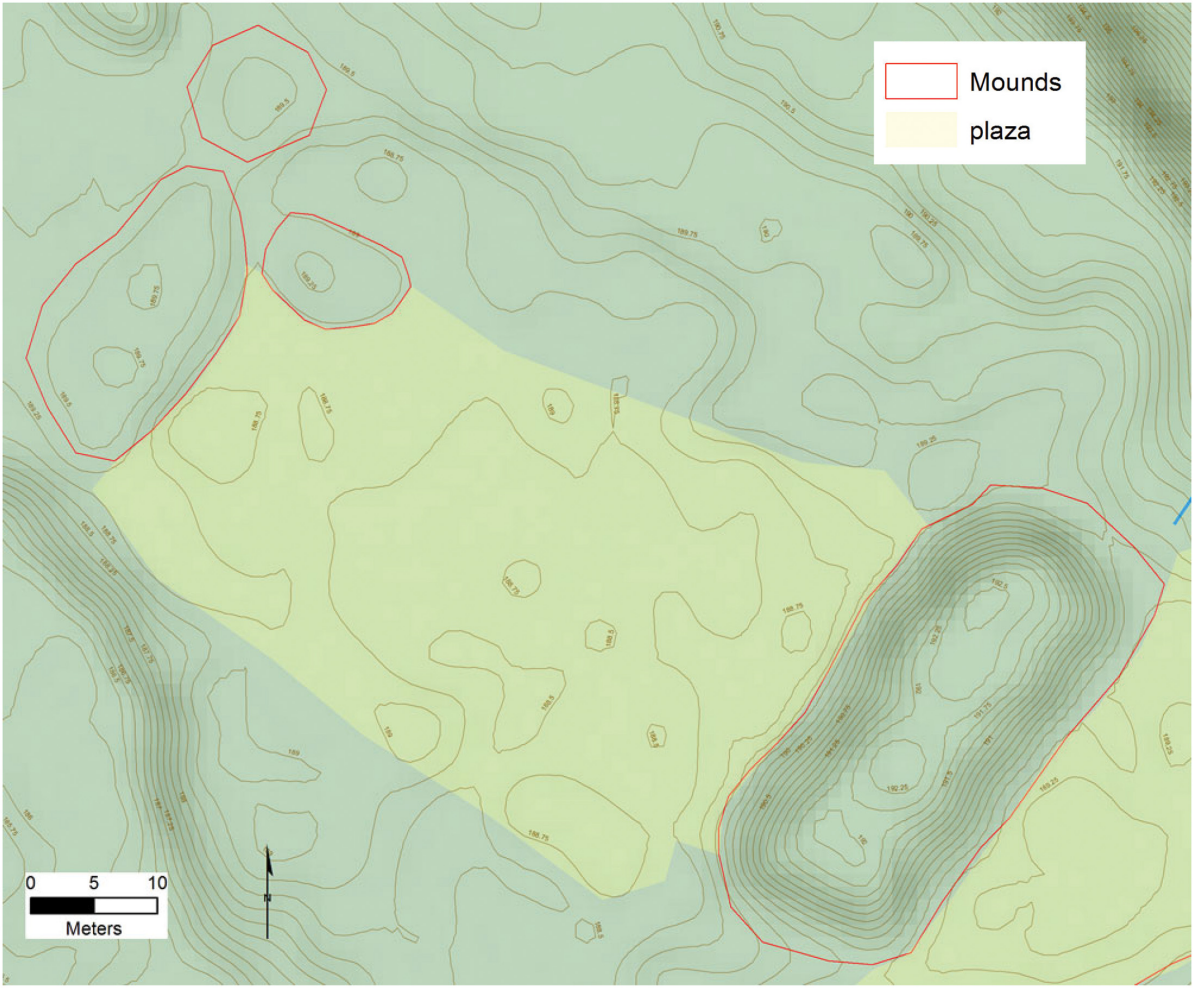
Example of a rectangular plaza configuration (Type B), taken from site 955 Jaguar. Digitized features are shown over a composite hillshade view taken from 16 different angles draped on a color shaded DSM with a resolution of 1 m/pixel. Contour interval is 25 cm. All visualizations created using high resolution aerial LiDAR.

All of the settlements we identified have one or more rectilinear mounds, composed of earth, and averaging 30 x 12 m, and 3 m high, along with numerous smaller circular mounds in various sizes and shapes, similar to others in NE Honduras [45,47,66,98–100]. These features appear both singly or in groups, arranged around plazas, and are most often associated with terraces and other landscape features. When mounds appear in groups, two basic spatial patterns were observed, with the most common formed by long linear platforms or embankments that enclose a large plaza in a square pattern which we refer to these as type A Plazas in our data dictionary (Fig 4). Access to the interior of the plaza comes from entrances at the four corners. At the site of *Jaguar* within the valley, stone stairways at the corners of the plaza allow access to the plaza center which may contain one or more small altars. Foundations for large buildings mark the tops of the surrounding mounds similar to the sites of Wankibila [65] and Las Crucitas de Aner [100]. The second spatial pattern is rectangular and anchored by two rectilinear mounds at the ends of a long plaza, which we reference as type B (Fig 5). The best preserved example of this pattern from Forteleza shows two small buildings at the top of each mound. In this configuration the sides of the plaza is most often open and access to the interior space is much less restricted.

Our data also show that the Valley inhabitants occupied a transformed and humanized landscape like other areas of the Americas [101]. Terraces are common, along with water control features that include ponds, canals, and channel diversion earthworks. The most visible landscape modifications are terraces represented by both narrow agricultural and wide habitation variants with the latter showing evidence of house foundations [102–104]. These are similar in some respects to low density urbanism systems recently documented for the Maya Postclassic [105–108]. In our initial investigation we were able to identify roughly 3 square kilometers of areas covered by anomalies that we feel represent terraced areas along with over 200 features that likely represent large individual terraces. Given the difficulty of seeing such features in the rugged topography and dynamic Valley environment this is likely a very low estimate.

**Fig 6:**
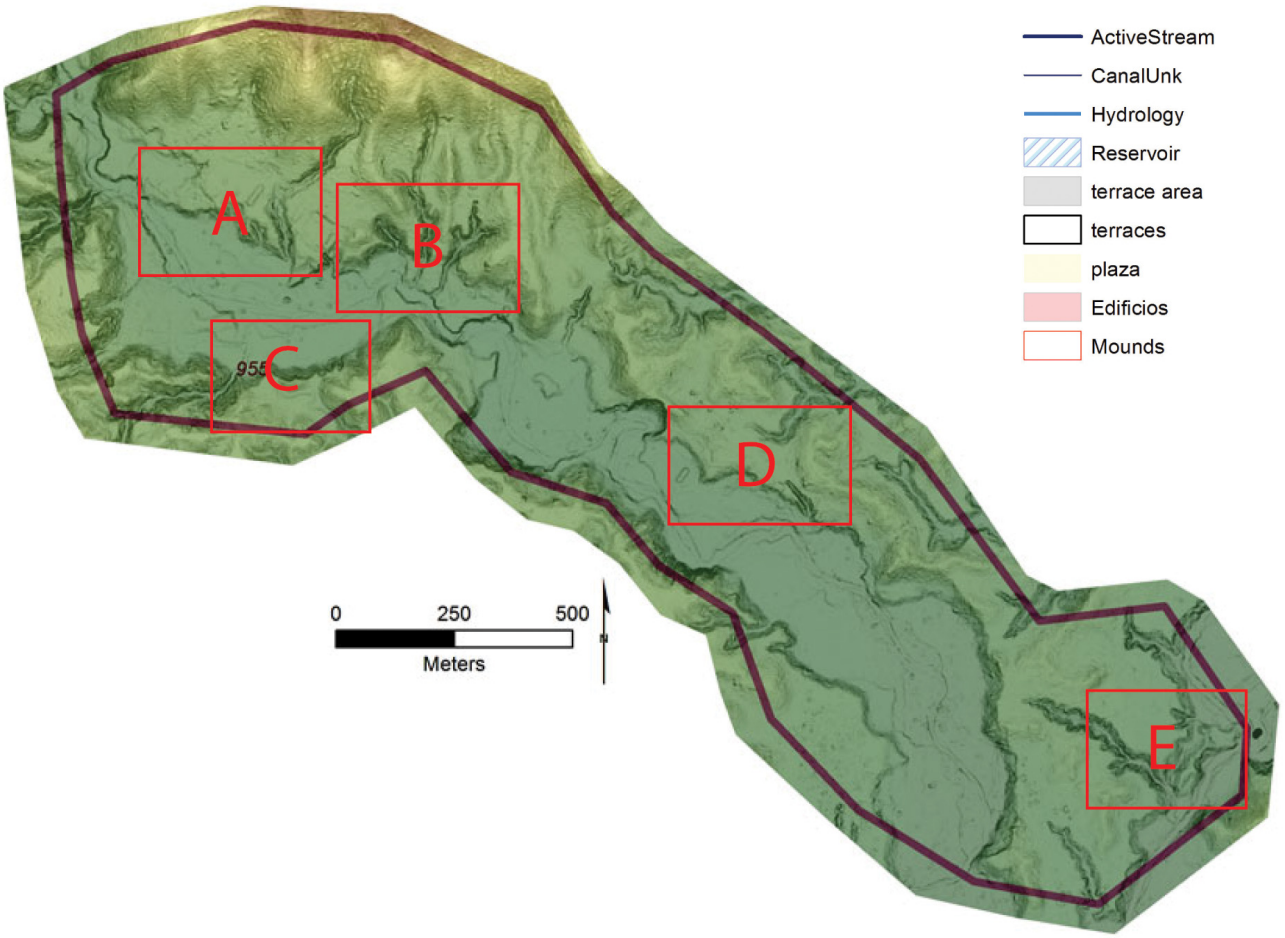
Key for Figs 7 and 12–14 showing the locations of the insets for the City of the Jaguar (site 955).

**Fig 7:**
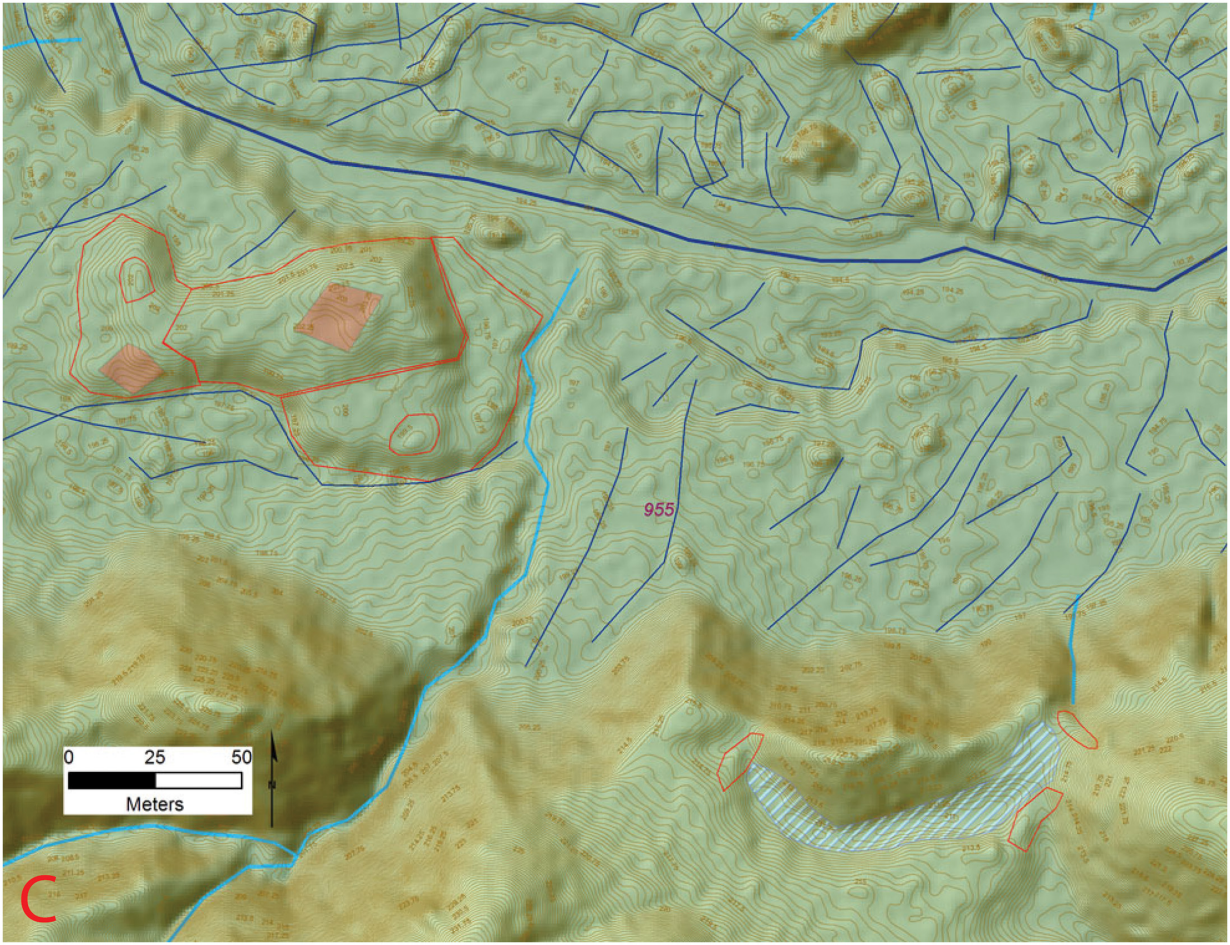
Example of a floodplain mound complex and associated upland reservoir (C) at site 955 (*la Ciudad del Jaguar)* within the V*alle de la Fortaleza* (T1). Location of section at the site and a legend are shown in Fig 9. Digitized features are shown over a composite hillshade view taken from 16 different angles draped on a color shaded DSM with a resolution of 1 m/pixel. Contour interval is 25 cm. All visualizations created using high resolution aerial LiDAR.

**Fig 8:**
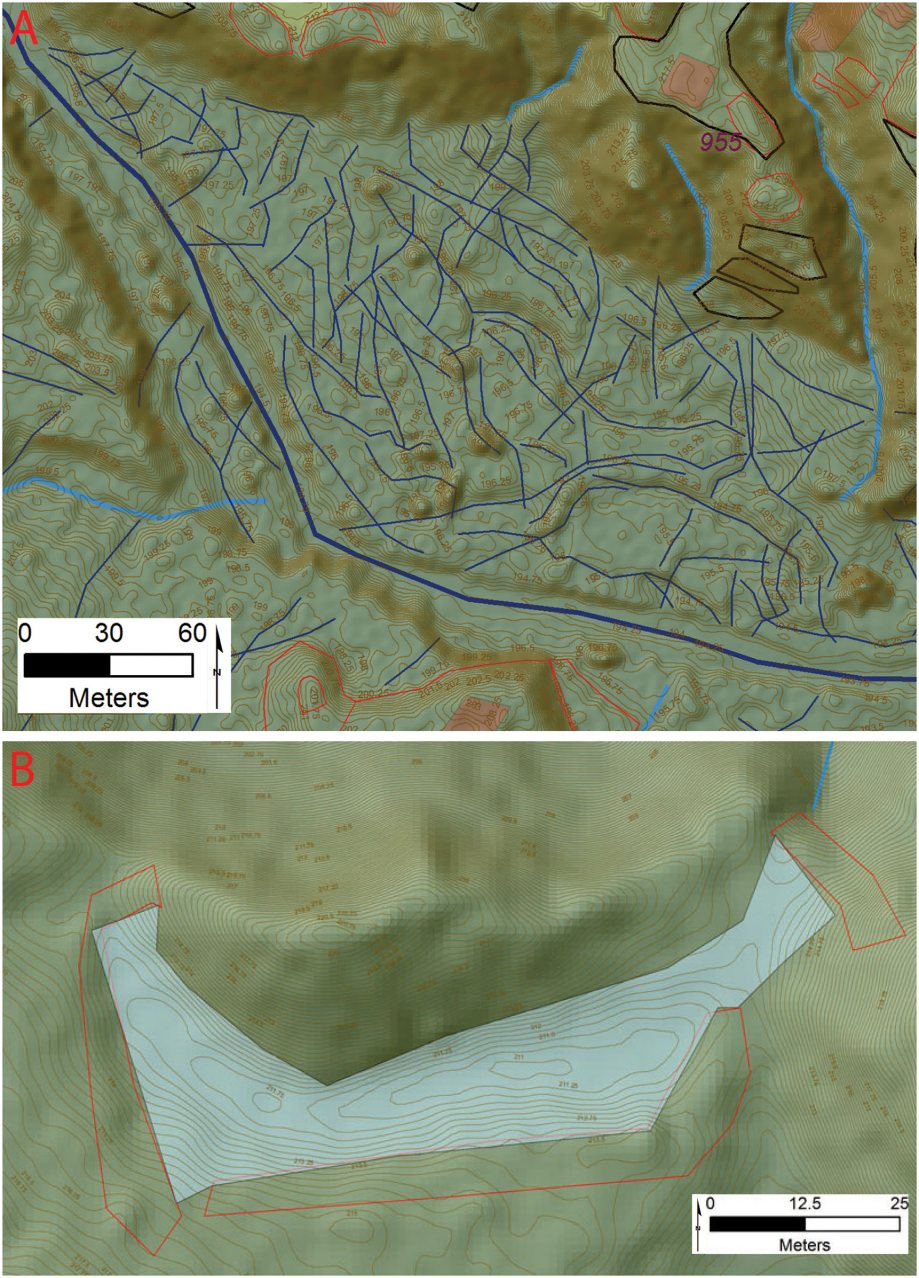
Water Control features from the Forteleza Valley. A) Possible canals associated with site 955; B) Reservoir from site 955. Digitized features are shown over a composite hillshade view taken from 16 different angles draped on a color shaded DSM with a resolution of 1 m/pixel. Contour interval is 25 cm. All visualizations created using high resolution aerial LiDAR.

The floodplain areas associated with several of the valley sites show large linear mounds composed of multiple overlapping platforms arranged in tiers topped by the foundations for structures and cultural debris (Figs 6 and 7). Given the presence of the floodplain settlements controlling the nature, course, and direction of the perennial drainages, they must have been a management priority. Abundant small channels are visible in the LiDAR data, arrayed in a dendritic pattern, and fastened at the upstream end by feeder canals (Fig 8A). Given their landscape position it is highly probably that these features are cultural and for water control but much future research is needed to confirm this hypothesis.

One reservoir, formed by a small valley perched roughly 20 m above the floodplain, was identified at *Jaguar* with an estimated volume of 1422 m³ as calculated in ArcGis (Figs 7 and 8B). Low spots around the exterior of valley were dammed with linear mounds drained by a sluice at the lowest end. Such features have been documented at Río Negro in [109] and are common in the Maya region [110] and perhaps related to unpredictable rainfall in the centuries leading to Conquest [40,111].

We identified 19 prehistoric settlements with architecture distributed throughout the Valley on diverse topographical settings (Fig 1) (S1 Supporting Information). In archaeology settlement systems are commonly characterized in terms of spatial organization and site size to better understand organizational relationships [112] and here we undertook such an analysis to better understand the comparative dynamics of the Forteleza occupation. For all tests conducted we used an area parameter of 26 km^2^ which represents the watershed area of the Forteleza Valley as determined in ESRI ARCGIS using the hydrology toolset on the full sample of 19 settlements identified during the LiDAR analysis.

Our analysis begin by examining relationships between sociopolitical complexity and the spatial distribution of settlements through a statistical point pattern analysis [113–118]. To evaluate the amount of clustering present in Forteleza settlements we first calculated the nearest neighbor statistic which determines the distance from the center of each settlement to that of the nearest adjacent settlement. The ratio of the mean of the distances compared to the mean of a set of points from a random distribution yields (R). An R value of 1 indicates a random distribution while a value greater than 1 shows a dispersed distribution and a value below 1 indicates clustering [119]. Statistical significance can also be determined (*p*) with a value below 0.10 indicating a significant result [118]

For the Forteleza sample of 19 settlements the observed mean distance is 838.6 m while the expected mean difference is 584.8 m yielding an R ratio of 1.43 indicating that settlements are dispersed across the Valley rather than clustered (Table 2). A *p* value of 0.000297 indicates less than a 1% chance that this pattern could be the result of random chance. Removing jaguar from the analysis reduced the R value slightly to 1.36 with a *p* value of 0.003284 but did not change the overall result. Thus this initial analysis shows a lack of hierarchical organization between settlements as is indicative of a middle range or chiefly society.

**Table 2:** Results of the nearest neighbor analysis. For all tests n = 19.

	Mean distance (m)	Expected distance (m)	Z score	R ratio	*p* value
All settlements	838.6	584.8	3.61	1.43	0.000297
W/out Jaguar	818.5	600.9	2.93	1.36	0.003284

The nearest neighbor statistic is one dimensional, however, in that it takes into account only the single nearest site [120–124] and can skew based on the areal extent of the region examined (8–12). A more sensitive measure of regional site distribution is Ripley’s K function/Multi-distance spatial cluster analysis (MDSCA) [125] which shows site clustering or dispersion over set distances [122–124]. As applied using ESRI ARCGIS MDSCA compares patterning from within the dataset to a simulated random sample for a measure of dispersion/clustering over specified distances. Output is illustrated as a scatterplot with distance intervals on the x axis and average distances on the y axis. Positive deviation from the expected random distribution indicates clustering while a distribution that falls below the expected regression indicates dispersal. Importantly this analysis can determine over what scales in space clustering or dispersion may occur and has become an important multiscaler means of settlement analysis in archaeology [122,126–129].

For our analysis we used a starting radius of 100 m that was increased in increments of 100 m up to a maximum distance of 4,000 m (40 distance bands) over 99 permutations (99 sets) to generate a random comparative sample. For an MDSCA simulation in ARCGIS a preferred size of 30 cases is necessary to ensure statistical significance. In our Valle de Forteleza sample contains only 19 meaning it would be difficult to get a significant result though the overall patterning is still meaningful.

**Fig 9:**
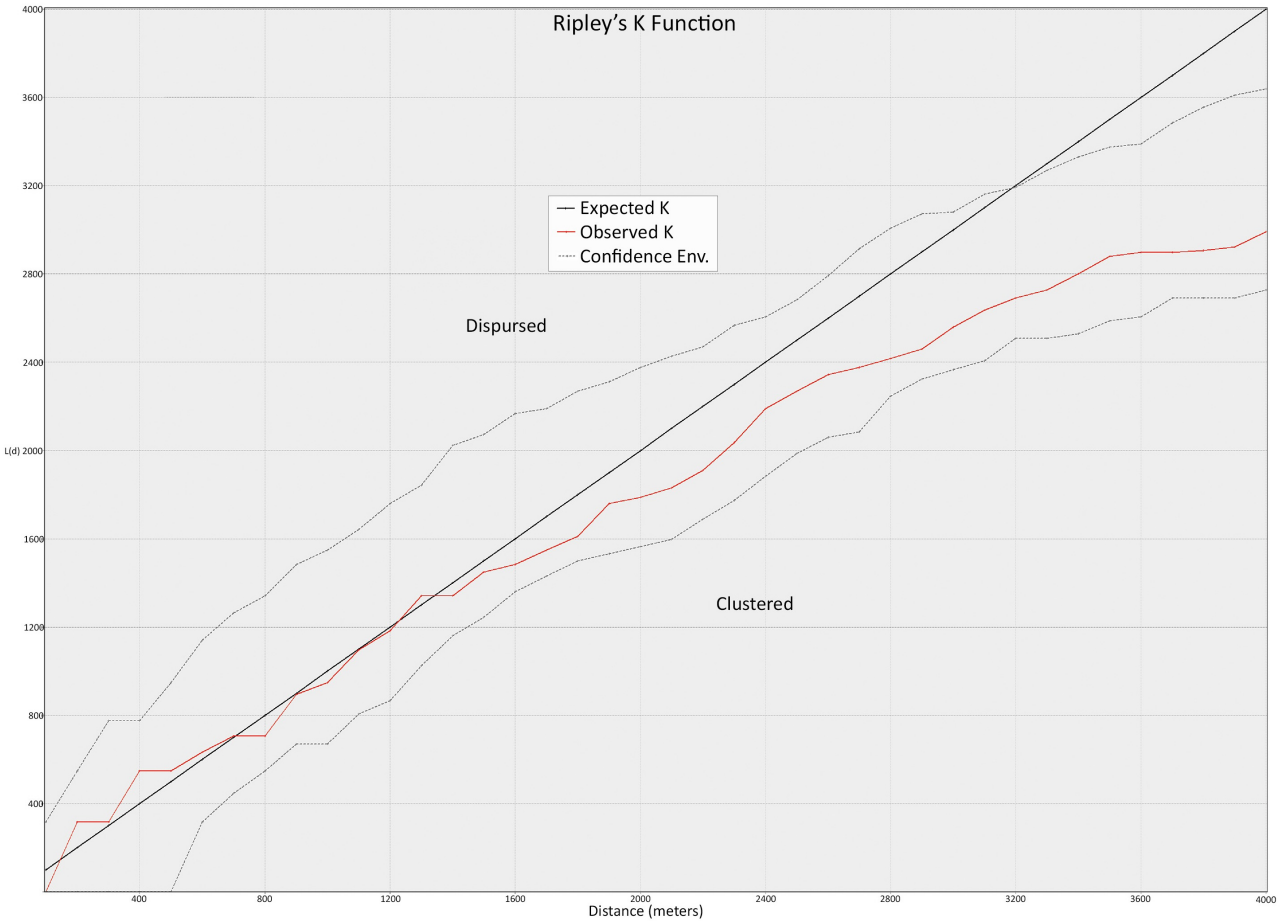
Ripley’s *K*/ Multi-distance spatial cluster analysis (MDSCA) for the 19 Prehistoric settlements identified in the Valle de Forteleza sample. The black ‘expected’ line shows the result of a simulated random distribution while the upper and lower dotted lines represent a 95% confidence interval. The red line shows the observed distribution for the Forteleza sample which shows increasing dispersion over distances of 1200 m.

The results of the MDSCA testing for the Valley sample confirms the nearest neighbor analysis in that at distances above 1,200 m the observed line clearly falls below the expected indicating a dispersed pattern of settlement (Fig 9). Given the small sample size however (>30 cases), the observed line falls between the upper and lower confidence interval indicating a result that is not statistically significant.

**Fig 10:**
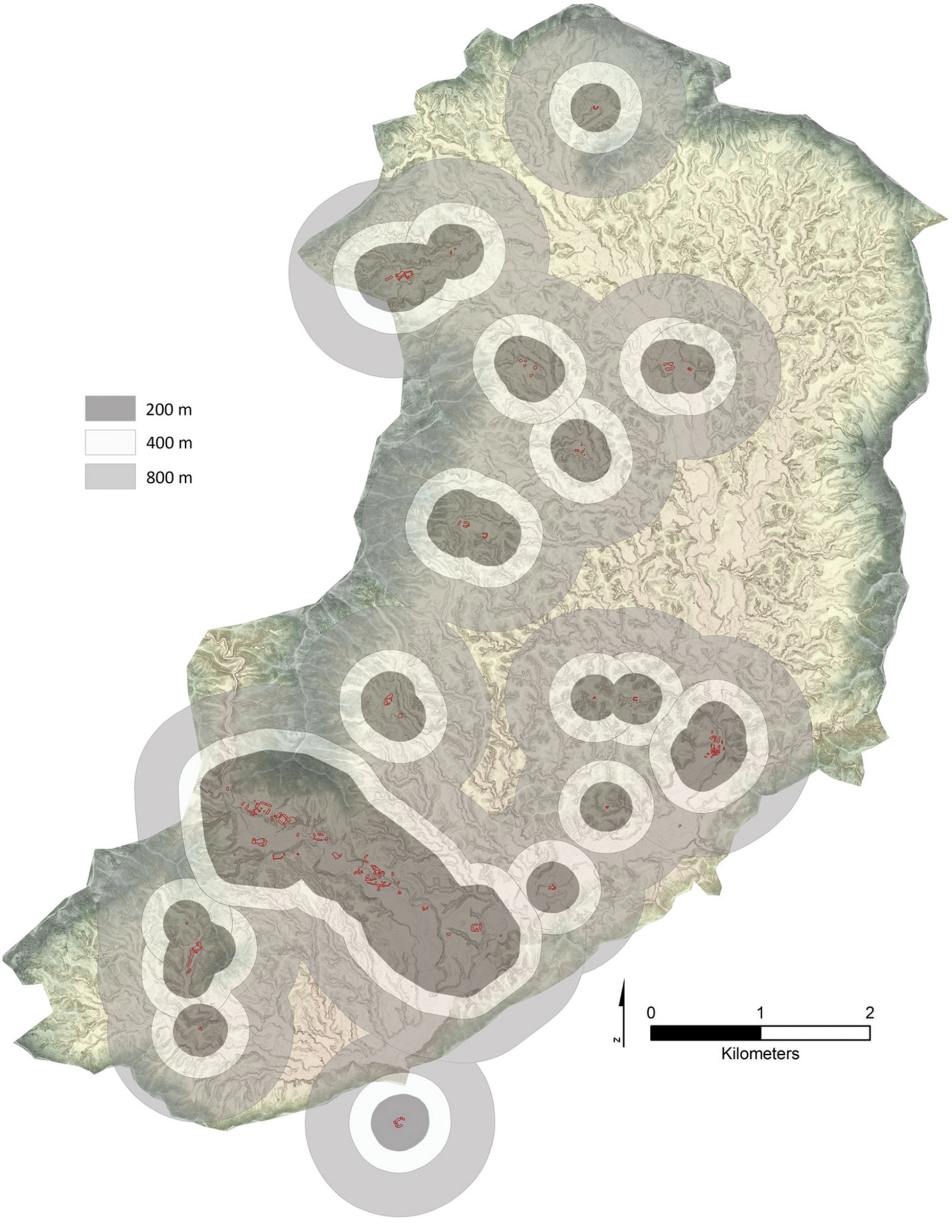
Buffers at 200, 400, and 800 m placed around the Fortelaza sites. Underlay is the sum of hillshades representing 16 cardinal points placed over a transparent DSM.

The data from the MDSCA does show some patterning between distances of 100–800 m which can be illustrated by using buffers placed around site polygons that originate at site centers for specified distances (Fig 10). These zones can serve as a rough measure of potential resource zones available to each settlement. Informed by the results of the MDSCA analysis we created buffers at intervals of 200, 400, and 800 meters in ESRI ARCGIS. Given the steep topography of the Valley overlap at the 400 m interval is clearly shown for most sites while at the 800 m interval significant overlap is shown. Thus some site patterning may be present, or was potentially developing, at the sub-regional scale prior to Valley abandonment. But the results from both point pattern tests conducted indicate a dispersed pattern of settlement location in which polities are maximizing available resource zones within a constrained regional pattern, like similar societies in Central and South America [130–135]

In terms of site location it has generally been thought for the Mosquitia region that settlement patterning during the Prehispanic period was riverine based given the rough topography and heavy modern vegetation and the resulting high overland travel times. The assumption that settlement location was largely determined by proximity to water course can be examined for the Forteleza settlements by conducting a Stream Order analysis in ARCGIS and comparing the distance to a high order stream segment to site locations. Following the methodology outlined in Tarboton [136] using the Stream Order Tool in ARCGIS we assigned a numeric order to all links in the stream network for the Forteleza DSM using the Strahler method [137,138]. This resulted in a stream network that ranged from 1–8. By combining the highest order streams together into a single shapefile (orders 6–8) we were able to calculate the near distance of the center of a polygon drawn around each digitized site to the nearest leg of a high-order stream.

**Fig 11:**
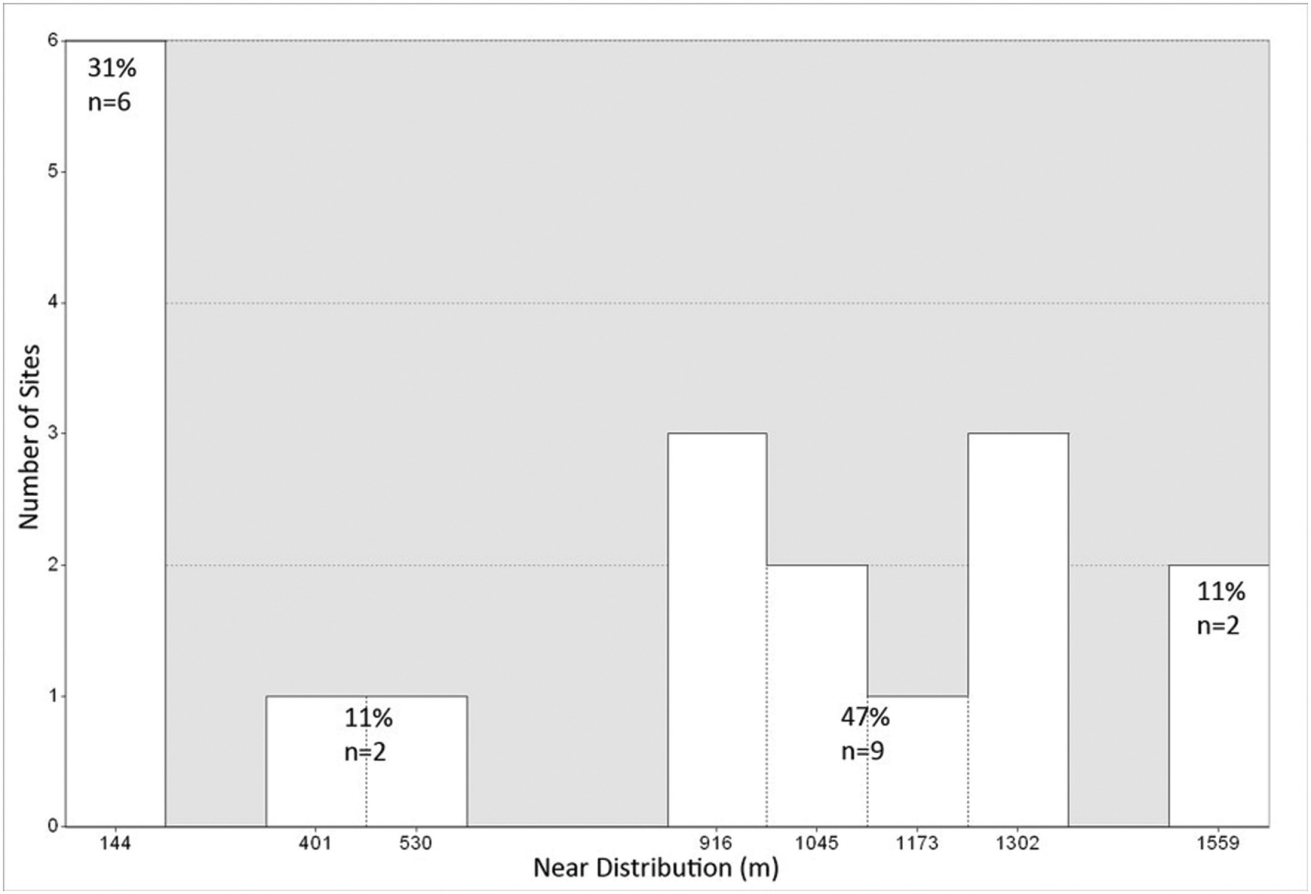
Histogram of the near distance from a high order stream segment to the center of a Forteleza settlement. Calculated using the near function in ESRI ARCGIS couple with a stream order analysis.

**Fig 12:**
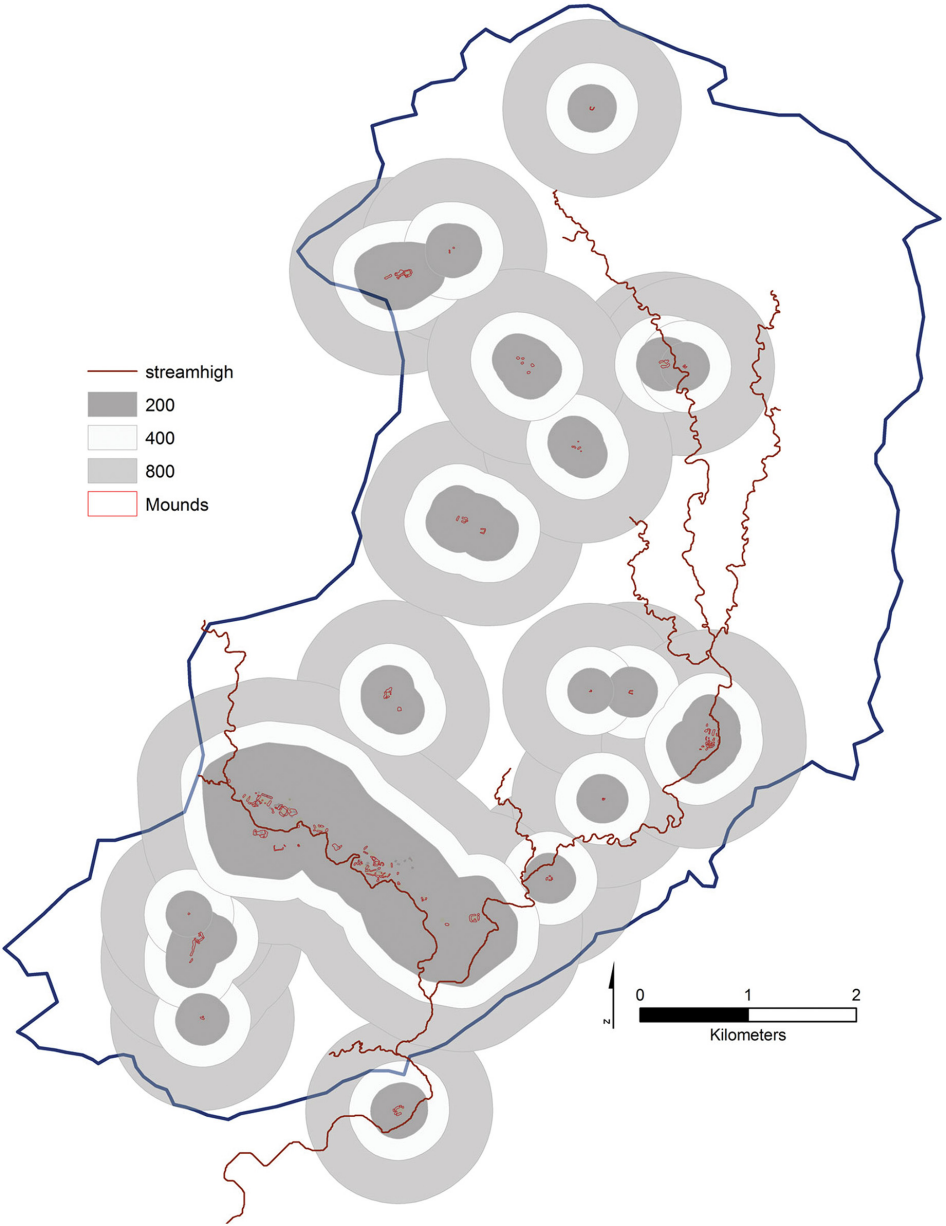
Buffers place around the Fortelaza settlements overlain on a visualization of the high order stream segments for the Valley. Blue line denotes the edge of the watershed.

Only six sites out of 19, accounting for 31% of the total number of Forteleza settlements, are located within 144 m of a high order stream. In contrast 47% of Valley settlements are located between 916–1,302 m of a high order stream segment while 11% are located over 1,559 m (Fig 11). This can be seen visually by overlaying the site buffers visualized in Fig 11 over the high order stream network (Fig 12). Thus over half of the Valley settlements are located over 900 m from a first order stream segment on upland areas.

We feel instead that site location within the Valley seems to be highly correlated with areas of low slope on the flatter Valley bottom. Though we lack soil or other data we feel it is a fair assumption that these areas are associated with richer sections of floodplain and piedmont soils. A visual assessment of a slope map generated using the ESRI ARCGIS slope tool with site location shows that flatter sections are preferred though this should be no surprise. We currently lack soil and other data that will likely show other associations with future work.

**Fig 13:**
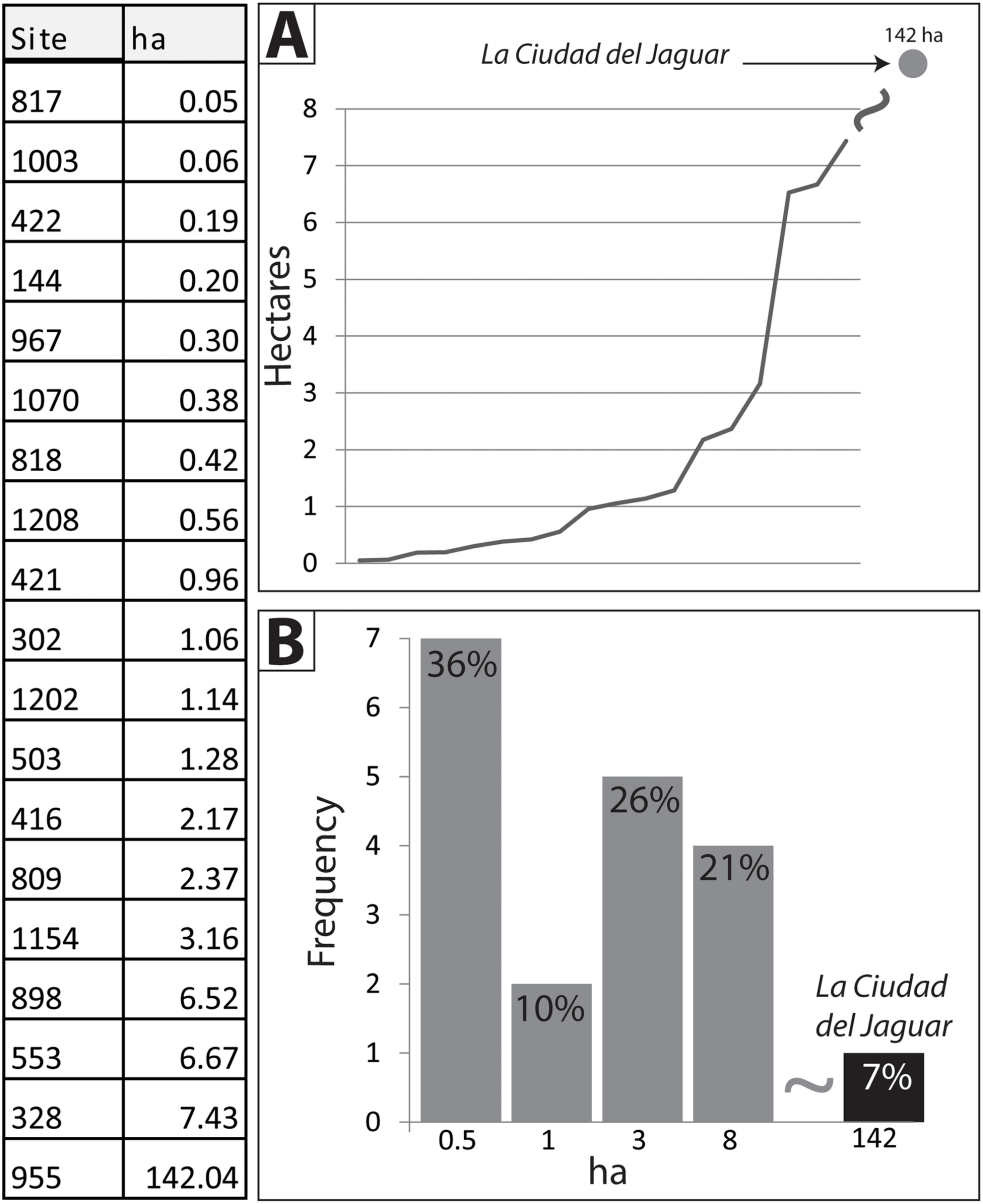
Core site areas in ha for the 19 settlements identified in the Valle de la Fortaleza. A) line plot of all sites showing size in ha; B) Histogram of site sizes.

The distribution of site sizes within a region has long been demonstrated to be a measure of socio-political organization for Prehistoric societies [112,139–144]. Here we examined the hierarchy of settlements within the valley by using histograms of site sizes coupled with a rank-size analysis. Using our LiDAR analysis we are able to only measure sites with mounded architecture and so it is likely that a smaller class of sites, composed of several houses or house groupings, cannot be discerned in our analysis. Through a visual examination of site sizes using a histogram we identified three rough clusters (Fig 13A and 13B).

**Fig 14:**
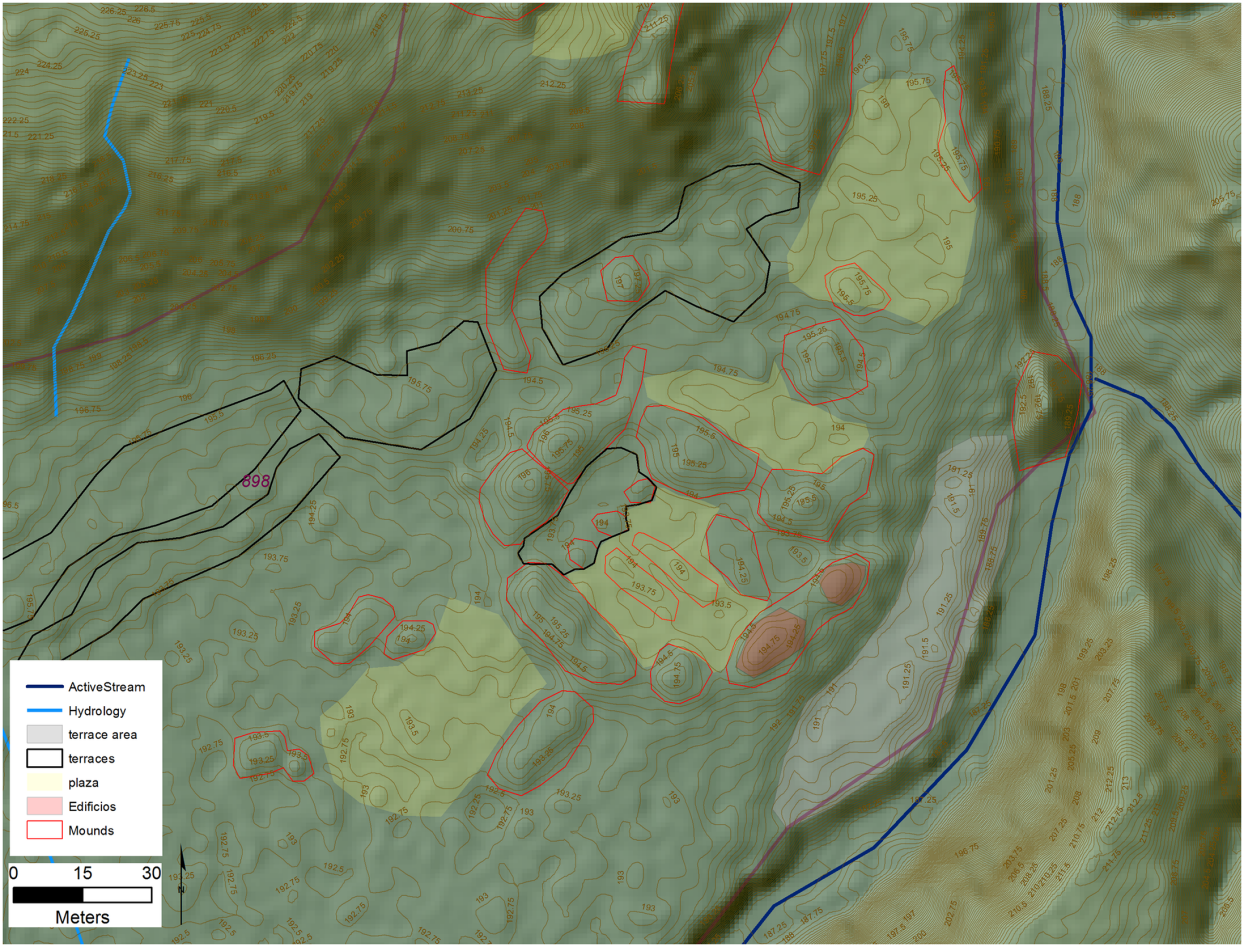
The central portion of site 898. Digitized features are shown over a composite hillshade view taken from 16 different angles draped on a color shaded DSM with a resolution of 1 m/pixel. Contour interval is 25 cm. All visualizations created using high resolution aerial LiDAR.

First, a group of settlements below 1 ha in size likely representing small hamlets composed of several households centered around plazas and one or more mounds (see Fig 4 for examples). Together this grouping comprises over 72% of the total number of settlements (19) identified in this analysis. A second cluster represents just three settlements over 5 ha in size likely representing villages and characterized by house foundations and public architecture such as plazas, large mounds, and other features. This middle tier is represented by clusters of architecture with core areas that are up to 8 ha in size. One example is site 898 located at the mid-point of the valley at the junction of several small streams (Fig 14). The core of 898 is roughly 6.5 ha in size and composed of a central Type A plaza group. Two additional groups to the north and south include smaller flanking mounds with large gaps on the perimeter. At the center of the main plaza are two parallel mounds that bear some of the hallmarks of a Mesoamerican-style ballcourt [145], similar to others documented for the Mosquitia [146].

**Fig 15:**
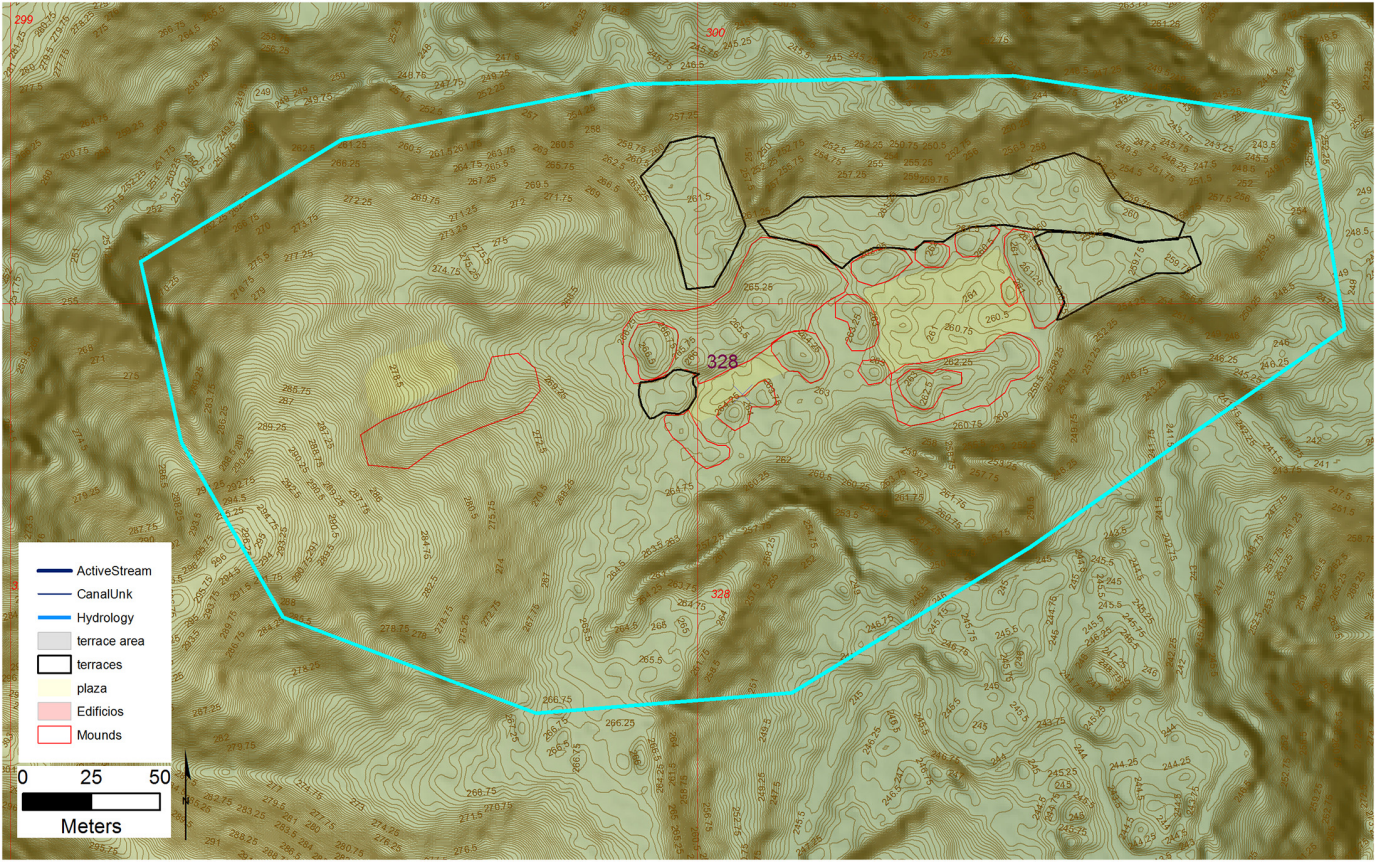
The main portion of site 328. Digitized features are shown over a composite hillshade view taken from 16 different angles draped on a color shaded DSM with a resolution of 1 m/pixel. Contour interval is 25 cm. All visualizations created using high resolution aerial LiDAR.

A second larger example is the site of 328 with a core area of 7.4 ha in size (Fig 15), and composed of three plaza groups, built into a natural bowl on an area of low bluffs. The upland placement, away from major drainages, is consistent with several other sites in the sample. The center of the site is dominated by a Type A patio complex with a second smaller and less complete grouping attached on the west side with a third small Type A complex present on the western edge of the site.

**Fig 16:**
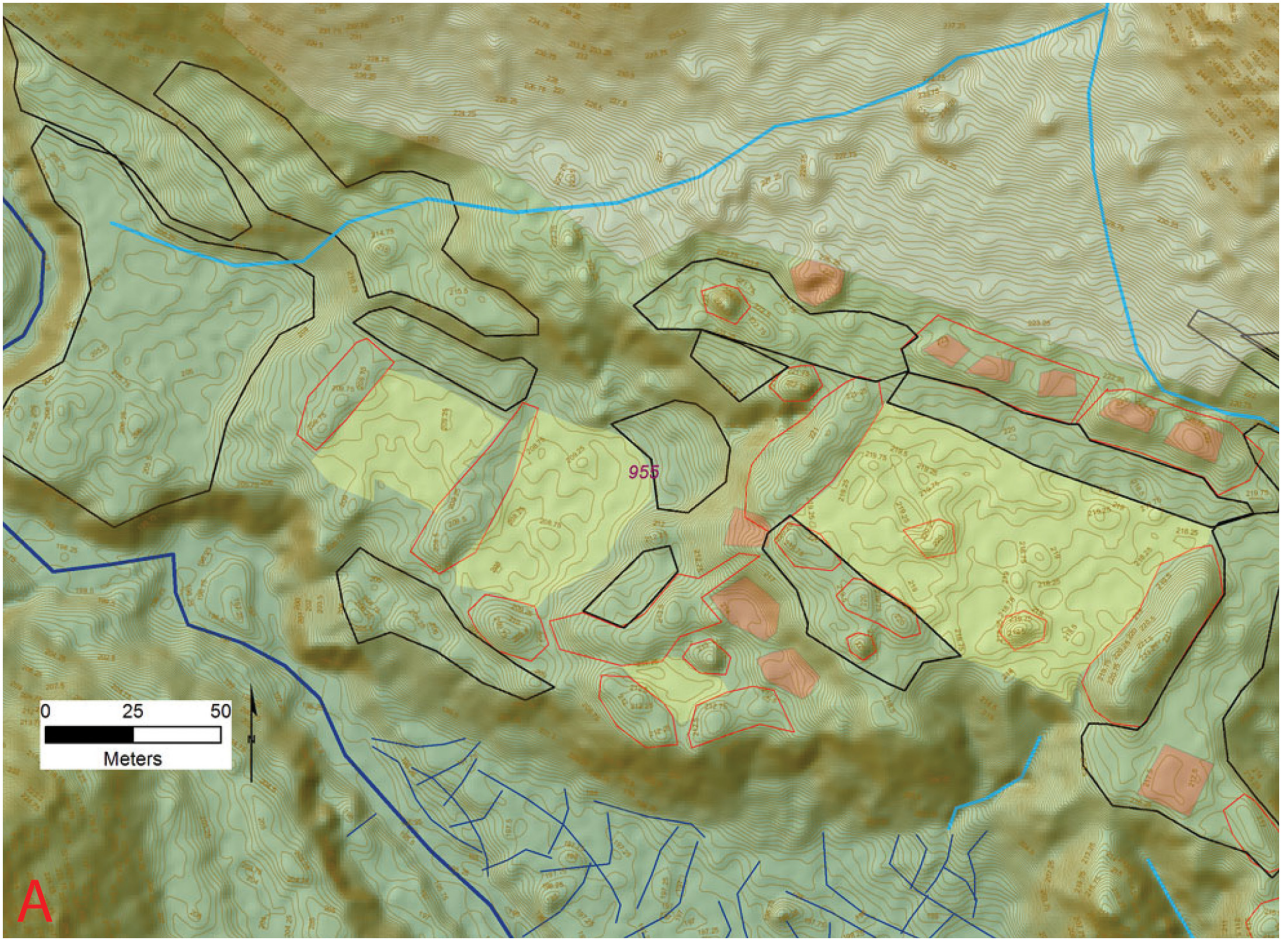
Eastern most section (A) of site 955 (*la Ciudad del Jaguar)* within the V*alle de la Fortaleza* (T1). Location of section at the site and a legend are shown in Fig 6. Digitized features are shown over a composite hillshade view taken from 16 different angles draped on a color shaded DSM with a resolution of 1 m/pixel. Contour interval is 25 cm. All visualizations created using high resolution aerial LiDAR.

**Fig 17:**
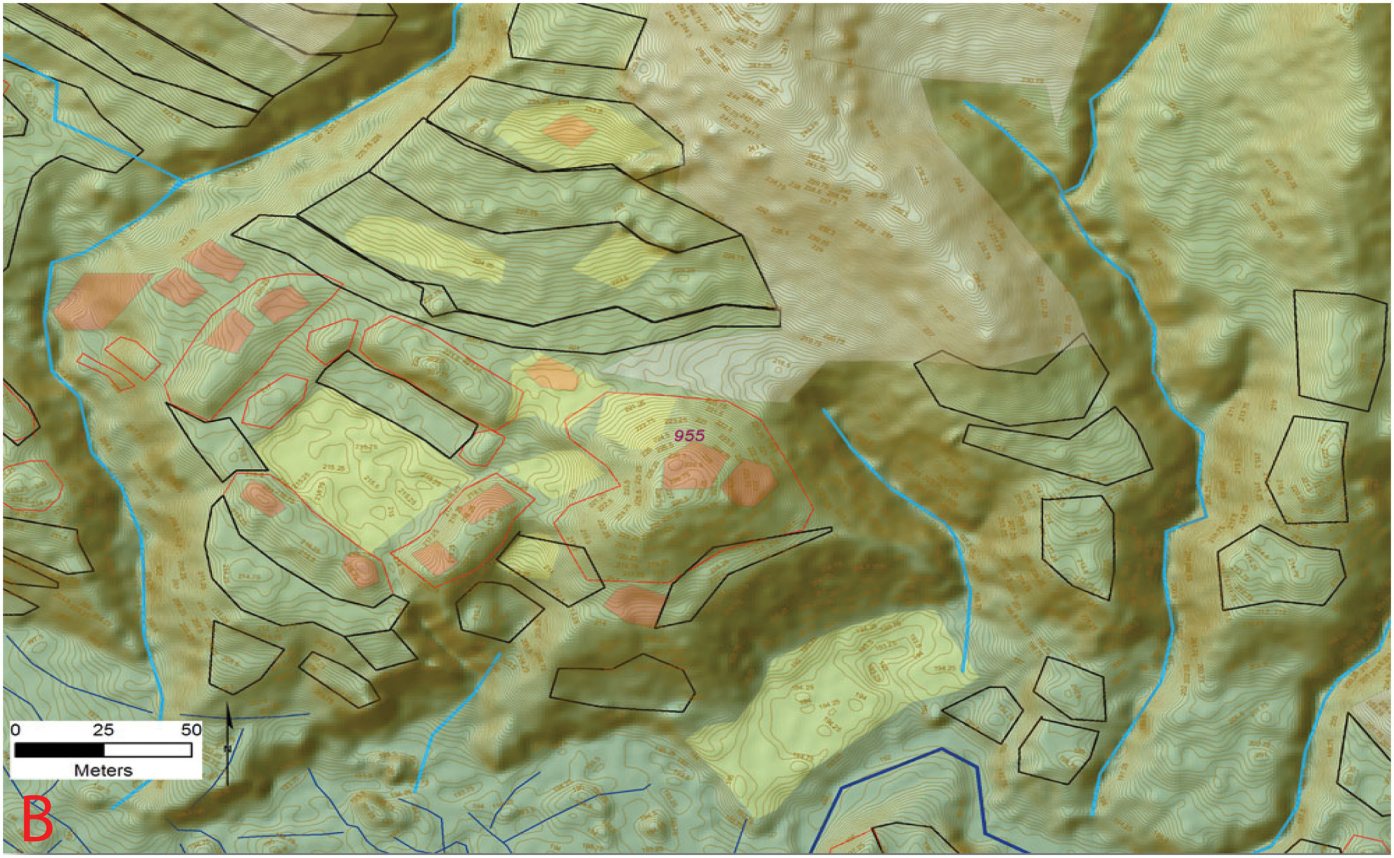
Eastern ‘cache’ location (B) at site 955 (*la Ciudad del Jaguar)* within the V*alle de la Fortaleza* (T1). Location of section at the site and a legend are shown in Fig 6. Digitized features are shown over a composite hillshade view taken from 16 different angles draped on a color shaded DSM with a resolution of 1 m/pixel. Contour interval is 25 cm. All visualizations created using high resolution aerial LiDAR.

**Fig 18:**
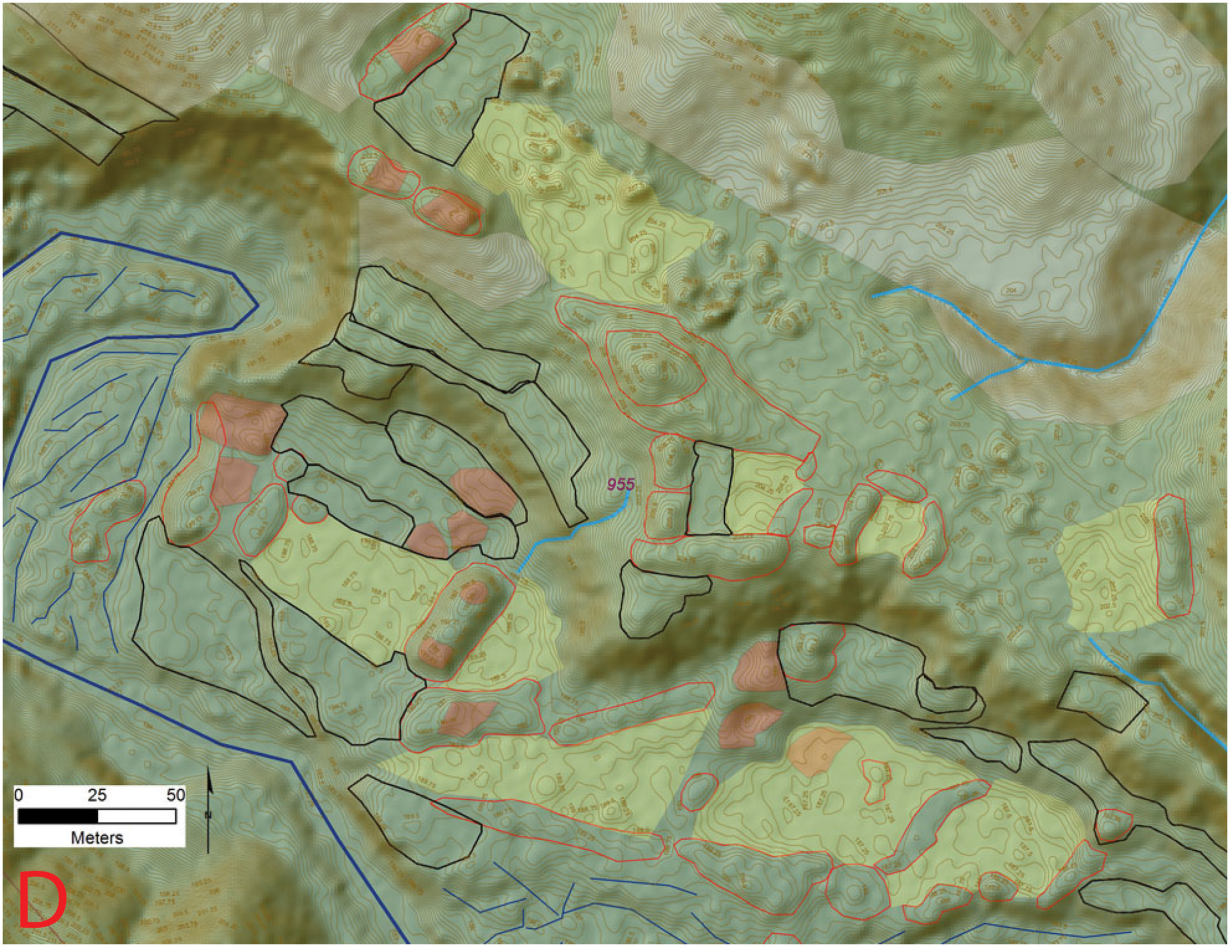
Central occupation area (D) at site 955 (*la Ciudad del Jaguar)* within the V*alle de la Fortaleza* (T1). Location of section at the site and a legend are shown in Fig 9. Digitized features are shown over a composite hillshade view taken from 16 different angles draped on a color shaded DSM with a resolution of 1 m/pixel. Contour interval is 25 cm. All visualizations created using high resolution aerial LiDAR.

**Fig 19:**
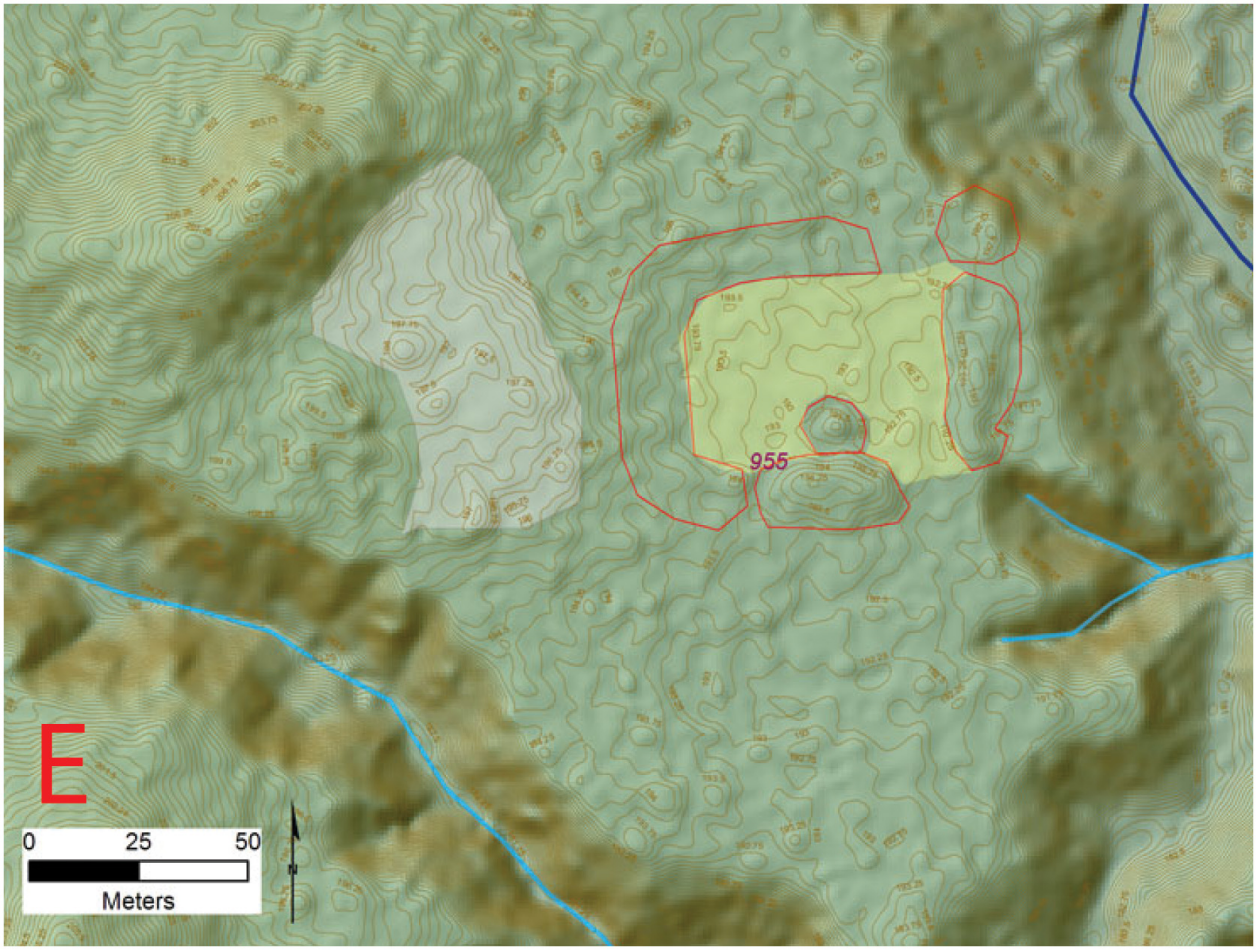
Eastern most edge of the settlement (E) at site 955 (*la Ciudad del Jaguar)* within the V*alle de la Fortaleza* (T1). Location of section at the site and a legend are shown in Fig 9. Digitized features are shown over a composite hillshade view taken from 16 different angles draped on a color shaded DSM with a resolution of 1 m/pixel. Contour interval is 25 cm. All visualizations created using high resolution aerial LiDAR.

A third and final group is comprised of the single site of Jaguar which is more than three times the size of the other sites within the Valley with a core area of 142 ha. The final settlement tier is represented by the *Jaguar* site (955), spread out for roughly 2 km along a series of bluffs that overlook the east/west drainage of the valley (Figs 6, 7 and 16–19) (S1 Movie). Running parallel to the stream is a jagged mountain range that divides the valley and provides an impressive backdrop to the settlement. With a monumental core of 1.42 km^2^ and an overall size that exceeds 3 km^2^, site 955 is significantly larger than the other sites within the valley and importantly, those known for the Mosquitia region.

*Jaguar* is composed of roughly ten plaza complexes averaging 50 meters on a side, arrayed sequentially along the bluff in two main clusters. Each complex faces the stream with a series of habitation terraces leading down to the active floodplain with smaller architecture composed of mounds, building foundations, wide terraces, and other features consistent with a residential occupation, on and around them. Behind each group are more residential and agricultural terraces depending on the steepness of the slope. The tops of the mounds immediately adjacent to the plazas are topped with large building foundations that likely represent temples or elite residences. The centers of the larger plaza groups contain the remains of one or more small structures that likely represent traditional-style altars.

At Jaguar the first cluster is located at the northwest sector of the city overlooking the adjacent floodplain which also yields evidence for occupation (Fig 16). The main portion of this zone is composed of several Type A patio groups with the space between the clusters occupied by roads and paths that lead back to residential terraces that give way to steep mountain slopes. The western edge of this portion of the site is dominated by a large earthen mound or pyramid with several small patios, enclosed on three sides, at the base. The eastern side of this feature (the side facing the plaza groups), shows evidence for a ramp or a staircase that leads to three small platforms at the summit of the mound with the foundations of several buildings on each.

During ground survey of the area, a cache of cultural material was discovered on the surface of one of the patios at the base of the possible staircase (Fig 17). The deposit includes numerous ground stone objects partially visible on the surface that can attributed to the Early Cocal Phase (A.D. 1000–1400) based on stylistic similarities to adjacent regions [45,61,63,64,147]. These include several stone bowls with effigies of a were-*Jaguar*, vultures, and other spirit animals, along with large grinding stones or seats with tripod bases known as *metates*. Though similar caches of objects have been noted in the region such as at Layasangi [148] and at Los Metates [66], the undisturbed nature of this cache is unique.

This deposit certainly represents a set of elite cultural items that were intentionally deposited in an important location of the ancient city. It is possible that they represent external symbols of power adopted by local elites as they were incorporated into the broader Cocal cultural sphere after the breakdown of the Classic Maya system [47,149,150]. They may also signal a period of greater connectivity for this region linked to the coast as was common during the Postclassic [45,151]. It is also possible that local populations during the Cocal period may have made many innovations on their own to create a similar but unique cultural style [152].

Around several of the plaza groups at site 955 stone altars were documented, represented by large flat slabs of rock, possibly shaped, and supported by three or more white quartz rounded boulders. These features have been identified elsewhere in the region though their function remains unknown [66,98,148]. At *Jaguar* the context is unique in that these features completely encircle the outer edge of major plazas at regular intervals suggesting a purposeful architectural function. The inner area of the plaza wall is also stepped so that the altars form the uppermost edge of the stepped interior of the plaza.

A second architectural grouping at the rough center of the *Jaguar* site is composed of two Type B plaza groups that are stacked above one another so the first occurs on the uppermost floodplain while the second is located on bluffs roughly 30 m above (Fig 18). At the top of each mound, the foundations for a single large rectilinear building were visible with possible stairs leading into the center of the plaza. These features formed the core of this central architectural cluster with smaller plazas and mound groups visible on the outer edges.

The Jaguar site lacks distinct boundaries or edges with mounded architecture and other cultural features extending away from the river systems into the adjacent uplands in an extensive fashion. Fig 19 shows one of these disconnected architectural groupings at the extreme eastern edge of the site. Though clearly connected to the central area of occupation shown in Fig 14 this plaza group also occupies a unique and private setting.

**Fig 20:**
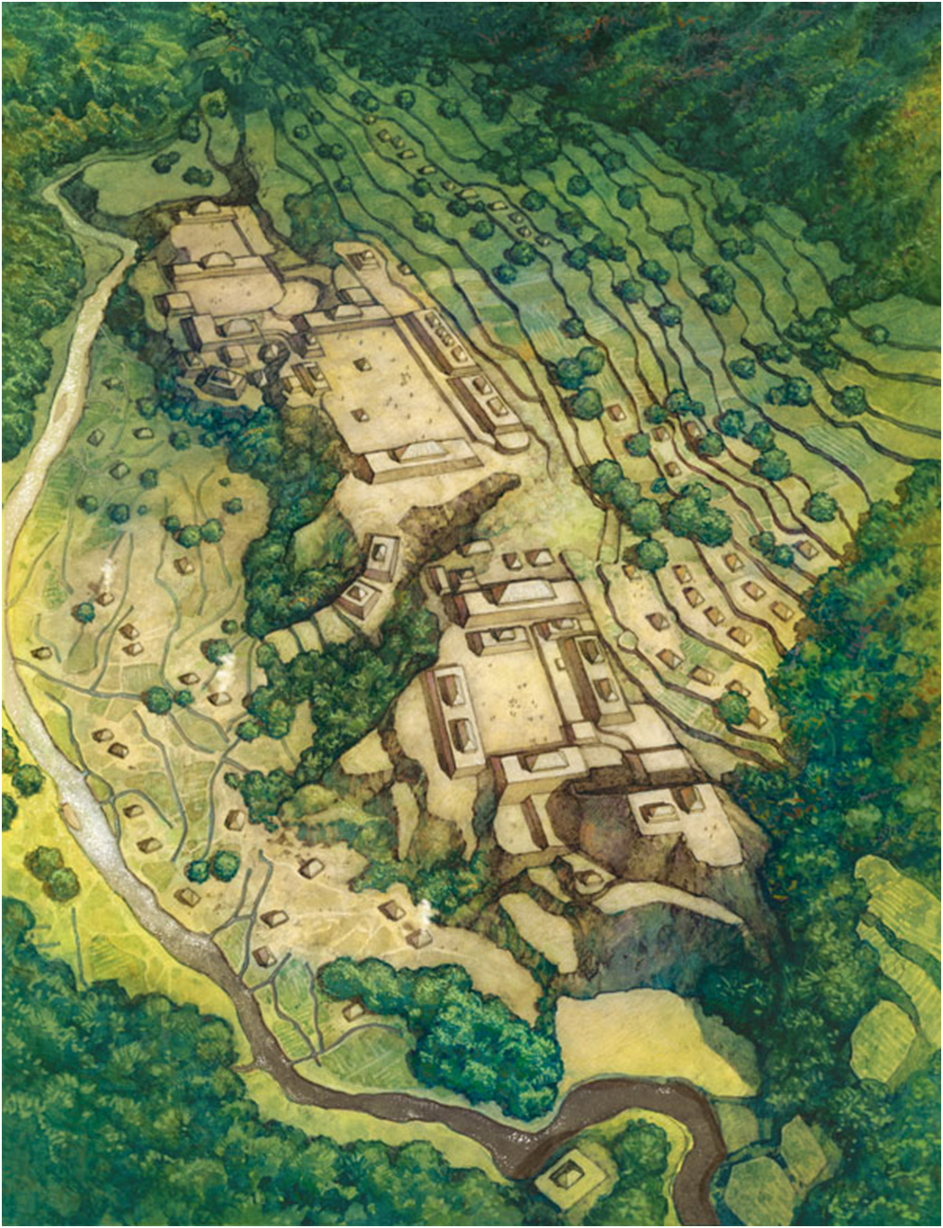
Artist’s reconstruction of one portion of site 955, *la Ciudad del Jaguar*, based on field-checked LiDAR data. View is looking roughly west and depicting site sections shown in Figs 10 and 11. Courtesy of Gregory A. Harlin/National Geographic Creative.

The overall topographical setting of 955 is similar in some ways to the Transitional Selin–Cocal phase site of *La Floresta* documented by Strong (1934). Like *Jaguar*, the main portion of *La Floresta* is located on bluffs overlooking the *Conquirre* River while the other side of the site is backed up against a steep slope. At *La Floresta*, however, the settlement is much smaller and is composed of a single Type A patio group, possible altars in the center, and smaller related mounds. *Jaguar* is significantly larger and more complex, though the similarities are intriguing (Fig 20).

Based on the size, spatial complexity, large-scale environmental manipulation, and landscape placement, we believe that site 955 is best characterized as a city [153–156]. Here we follow the broad definition outlined by Trigger [157] and also M.E. Smith [155] who view a city as the venue for specialized activities that impact a wide hinterland. Though the ultimate resolution of this designation must await excavation and long-term intensive investigation we rely on several lines of evidence in the interim. First the altars, overall scale of the plazas, presence of the earthen pyramid at the center of the site, and other unique features of the plaza complexes suggest specialized ritual functions that are not present at other sites within the Valley, or perhaps even the region. Next, we see evidence for social differentiation in the size and placement of building foundations, especially differences in those on platform mounds at the edges of plazas, and those on the surrounding terraces. We also see functional differences between and within plaza complexes that we feel imply spatial distinction. Some of these could be considered sub-divisions within the settlement structure similar to neighborhoods (Smith 2010). Also the scale of the built environment in terms of water control, terracing, and connective features belies landscape management and organization at a large scale. We understand this claim may be controversial but feel that future research will provide additional evidence to bolster our assertion.

The number of levels in a regional site hierarchy is an important measure of the scale and centralization of a polity. It is generally recognized that a two-tiered arrangement is indicative of a chiefdom level of organization while three or more tiers is generally associated with a State [139,158–160]. The hierarchial arrangement of Forteleza settlements is nominally two-tiered with the City of the Jaguar forming a third tier as a primate center. Given the small size of the Valley settlements and the lack of spatial integration as demonstrated by the point pattern analysis we do not feel a State-level society is indicated. Instead it seems likely that two distinct settlement systems are superimposed over one another represented by a two-tiered internally generated Valley system and a second, externally generated, system that promoted the Jaguar site as an extra-regional center. The two tiered system would be similar to others that have been documented for adjacent areas of Central America [150,161–163]

This can be shown by performing a rank-size analysis on the Valley settlement system. Rank size has long been used as a method of exploring settlement hierarchies in archaeology [113,139–141] following the idea that the population of a settlement should be inversely related to its rank in a regional hierarchy [164]. When plotted on a logarithmic scale the resulting plot yields a straight line from upper left to lower right and known as ‘log-normal’ [140,141]. Deviations from a straight pattern can be described by shape and are associated with different modes of hierarchial organization [164].Our analysis was performed using the RankSize simulation program version 3.2 [165]

**Fig 21:**
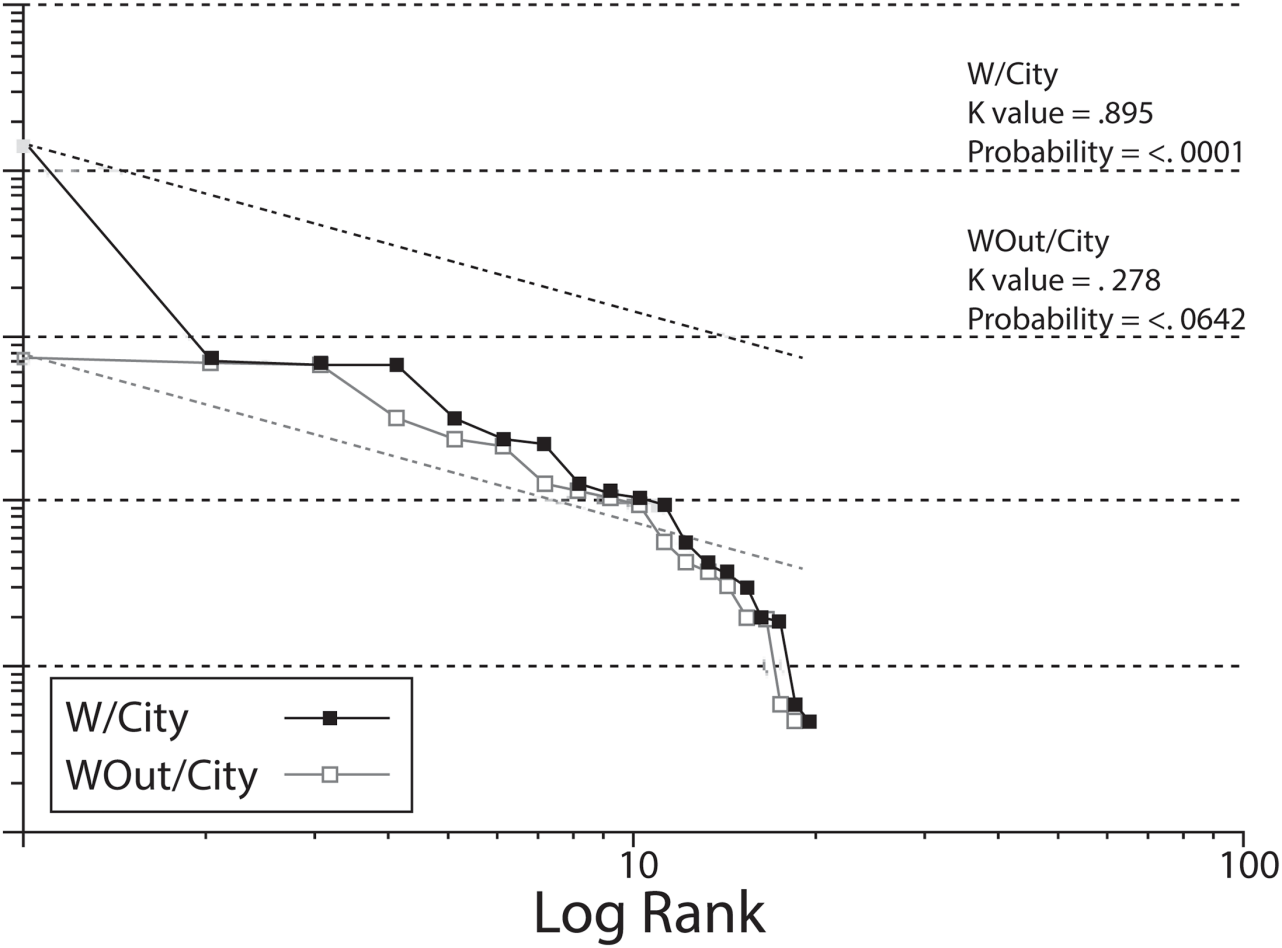
Plot of Rank-size analysis of valley sites with the city included (black) showing a primo-convex pattern, and the city excluded (gray), showing a double or two tailed pattern.

Our analysis yields a two tailed primo-convex pattern with a K value of .895 indicating a significant deviation from the rank-size rule [113,140,141] (Fig 21). This type of distribution commonly occurs when an external polity, represented by a large site in the upper part of the curve, imposes control over a network of less integrated smaller sites, appearing as the lower part of the distribution [142,144,166]. Interestingly, when Jaguar is removed from the analysis, a double convex curve is formed with a K value of .278 indicating a more integrated distribution that is similar to prehistoric Europe in which a localized settlement hierarchy was not incorporated into broader systems of settlement [167]. This situation is similar to others in which a primate pattern has evolved from something other than the development of social complexity [168].

We envision three historical trajectories that could have resulted in the distinct primo-convex settlement distribution. First, it has been well documented that during the Mesoamerican Terminal Classic, population shifts resulted in migration and the reorganization or formation of polities and settlements [39–43]; cf. [169]). Thus, some of the population increase documented for the region during the Cocal period could have resulted in the formation of *Jaguar* [66].

A second alternative focuses on a late extension of influence by one of the coastal region ‘super chiefdoms.’ The formation of *Jaguar* could represent a late extension of control by chiefdoms documented by the Spanish which include Taguzgalpa, Naco, Chapagua, and Papayeca [45–47]. None of these chiefdoms has been confirmed at a known archaeological site, so this explanation remains to be tested.

A third possibility relates to a process of synoikism, as has been documented for other areas of Mesoamerica [170–173]. The topography of the valley served as a defendable, fortified location and if there were expansionist polities during the Cocal phase, then *Jaguar* could represent a place of unification to better resist coastal incursion. Alternatively, this same process could have occurred as indigenous populations sought places of refuge from European Conquest.

These results are hypothetical and contingent on a fuller understanding of the chronological sequence of settlement awaiting systematic excavation and dating. Stylistically, however, the artifacts found on the surface of 955 date to the Early Cocal phase and so, by extension, does the architectural patterning at the site. This dates the *Fortaleza* settlements to the centuries preceding European contact during the Postclassic period. The degree of landscape modification and the overall settlement density, however, certainly hint at a valley occupation that has greater time depth.

## Conclusions

Conducting regional-scale archaeological research in tropical regions has long been a daunting and often impossible task. Here, we have been able to document the complete pattern of settlement for a critical river valley in a scientifically unexplored region. Our work clearly shows that though today the area is a tropical wilderness, in the past it was a dense mosaic of human settlements embedded within an engineered environment. The fundamental settlement unit within this system was a large plaza group with historical antecedents that are distinct from other Mosquitia region occupations. Our data also show evidence for a strong connection and perhaps rotational control at one time by coastal peoples connected by circum-Caribbean groups. Indeed, it may be that late in the prehistoric sequence, this critical area in the headwaters region of the Río Pao formed a strategic zone of control for the late Taguzgalpa polity. It is also possible that the settlement of this region is tied to the societal perturbations associated with the European Conquest of the Americas.

## Supporting Information

S1 Movie Animation of the central area of the City of the Jaguar (Site 955). Major mounds are digitized in red. Vegetation represents a color-shaded version of the point cloud data. Underlying color shaded DSM has a resolution of 1 m/pixel.
(MOV)

S1 Supporting Information Settlement maps for the 19 sites identified in this research. Each map shows digitized archaeological and topographical features. Digitized features are shown over a composite hillshade view taken from 16 different angles draped on a color shaded DSM with a resolution of 1 m/pixel. Contour interval is 25 cm. All visualizations created using high resolution aerial LiDAR.
(PDF)
