# Supplementary material for: Identifying Ancient Settlement Patterns through LiDAR in the Mosquitia Region of Honduras
Source: PLoS One. 2016 Aug 25;11(8):e0159890. doi: 10.1371/journal.pone.0159890 (PMC4999160; doi:10.1371/journal.pone.0159890)
Supplement: S1 Supporting Information — Each map shows digitized archaeological and topographical features. Digitized features are shown over a composite hillshade view taken from 16 different angles draped on a color shaded DSM with a resolution of 1 m/pixel. Contour interval is 25 cm. All visualizations created using high resolution aerial LiDAR. (PDF) [file pone.0159890.s002.pdf]

S1 Supporting information. **Settlement maps for the 19 sites identified in this research.** Each map shows digitized archaeological and topographical features. Digitized features are shown over a composite hillshade view taken from 16 different angles draped on a color shaded DSM with a resolution of 1 m/pixel. Contour interval is 25 cm. All visualizations created using high resolution aerial LiDAR.

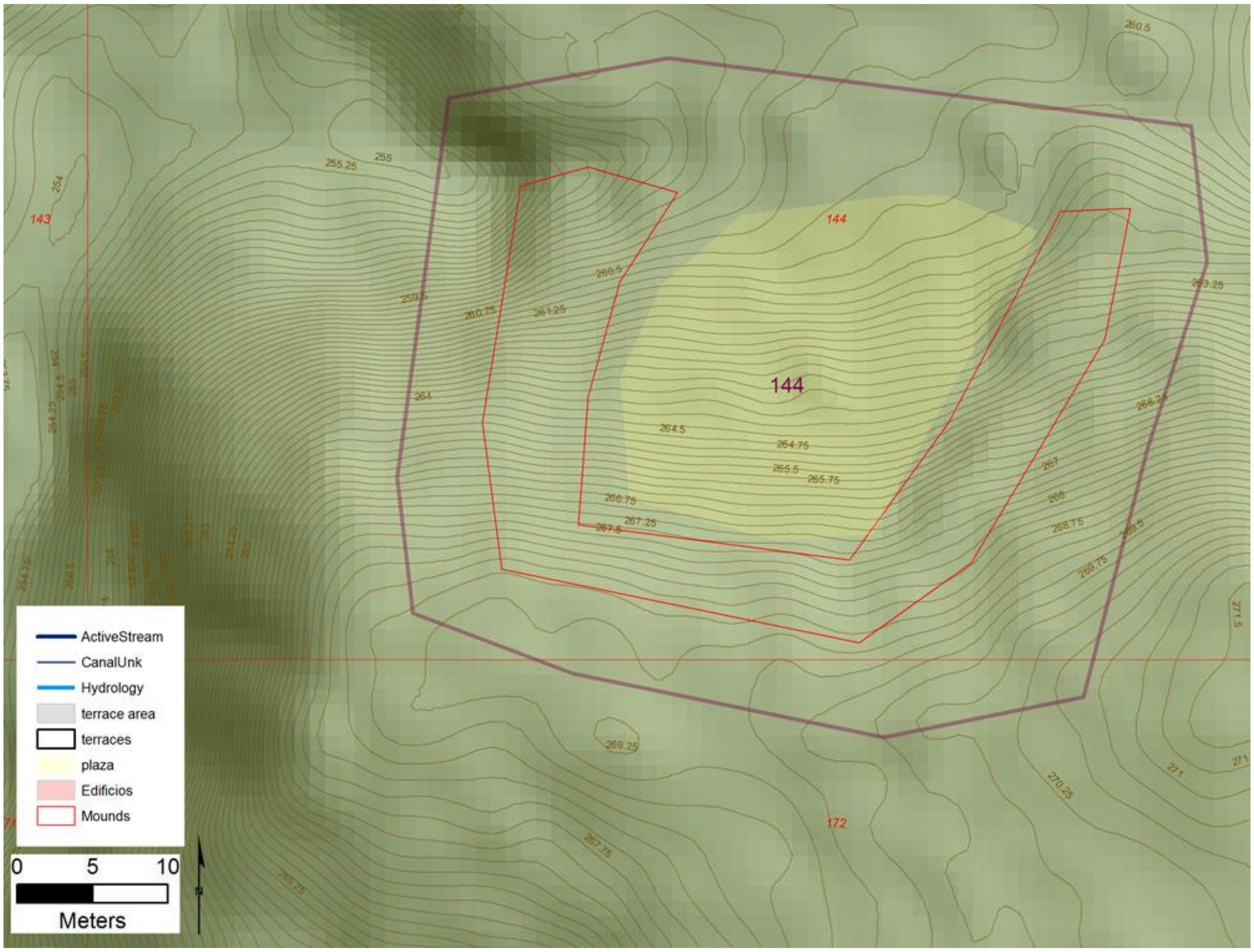

Site 144

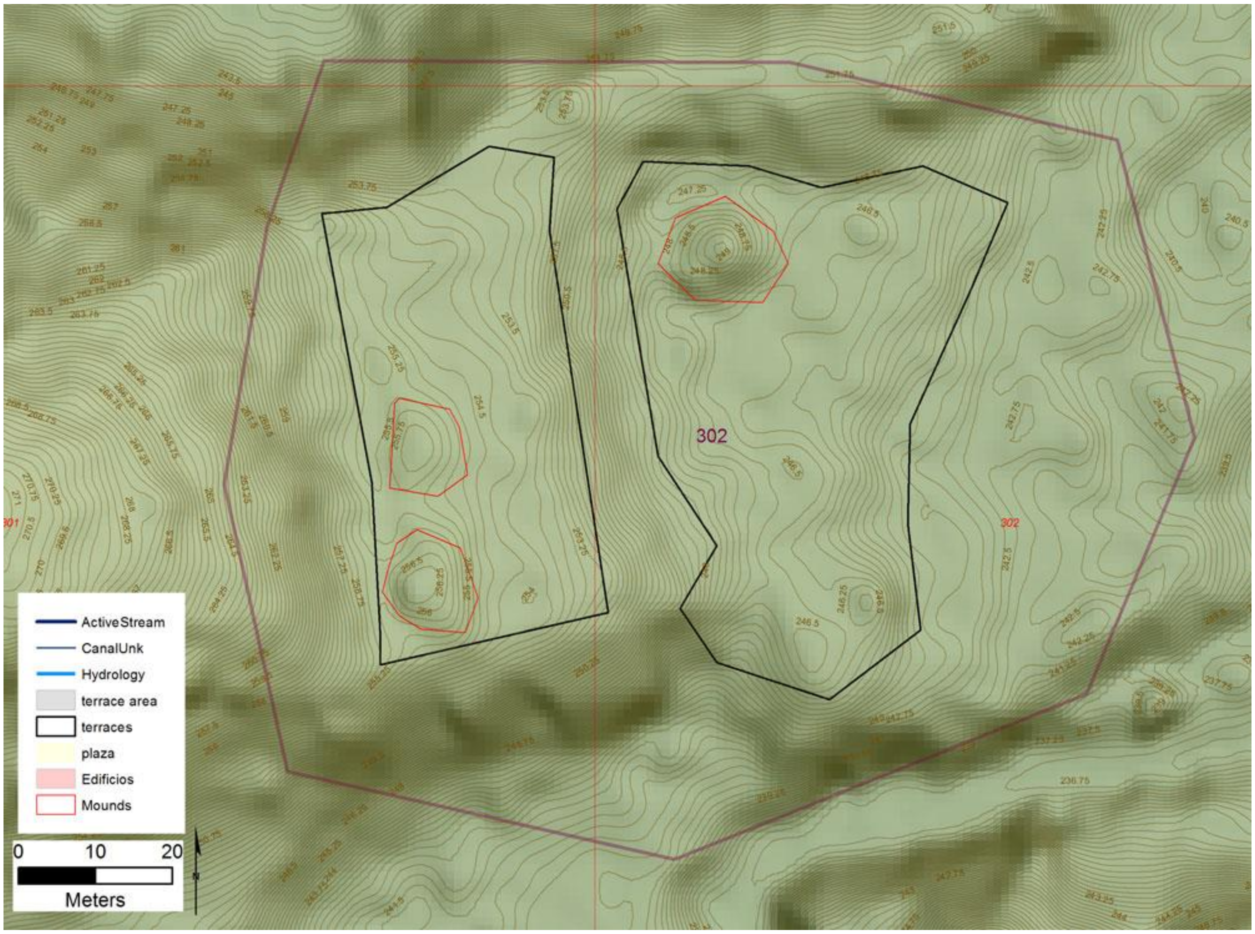
302
ActiveStream
CanalUnk
Hydrology
terrace area
terraces
plaza
Edificios
Mounds
0
10
20
Meters

Site 302

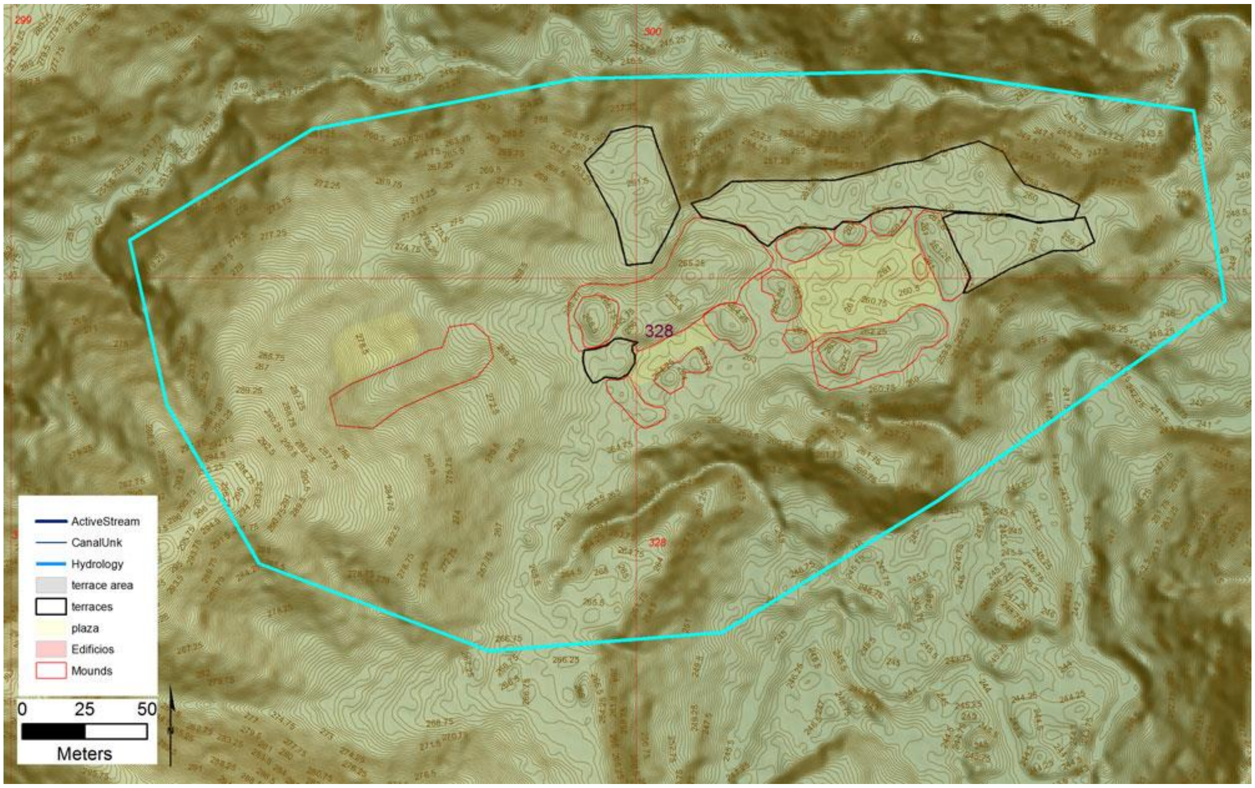
ActiveStream
CanalUnk
Hydrology
terrace area
terraces
plaza
Edificios
Mounds
0
25
50
Meters
328

Site 328

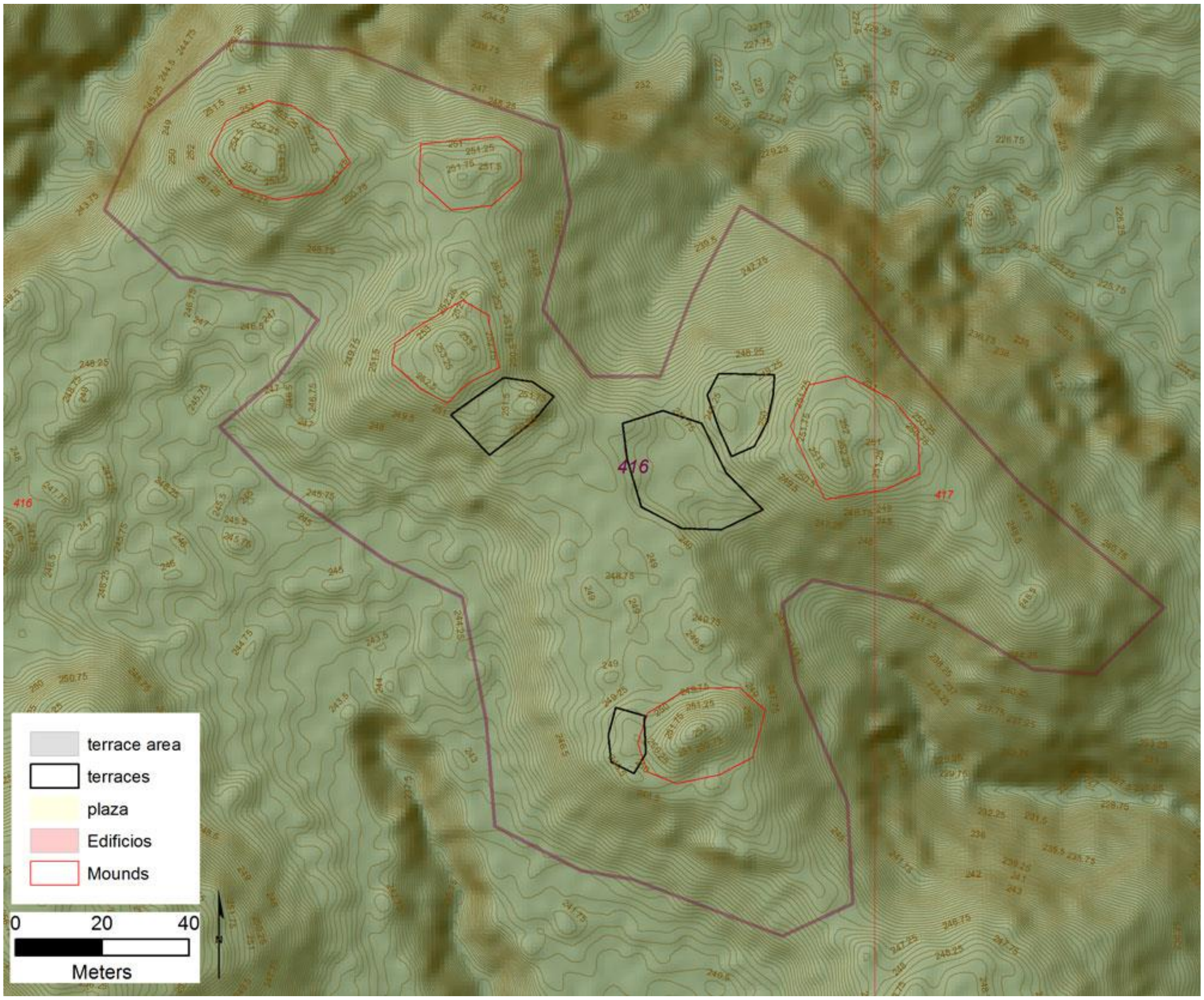

416
417
terrace area
terraces
plaza
Edificios
Mounds
0
20
40
Meters

Site 416

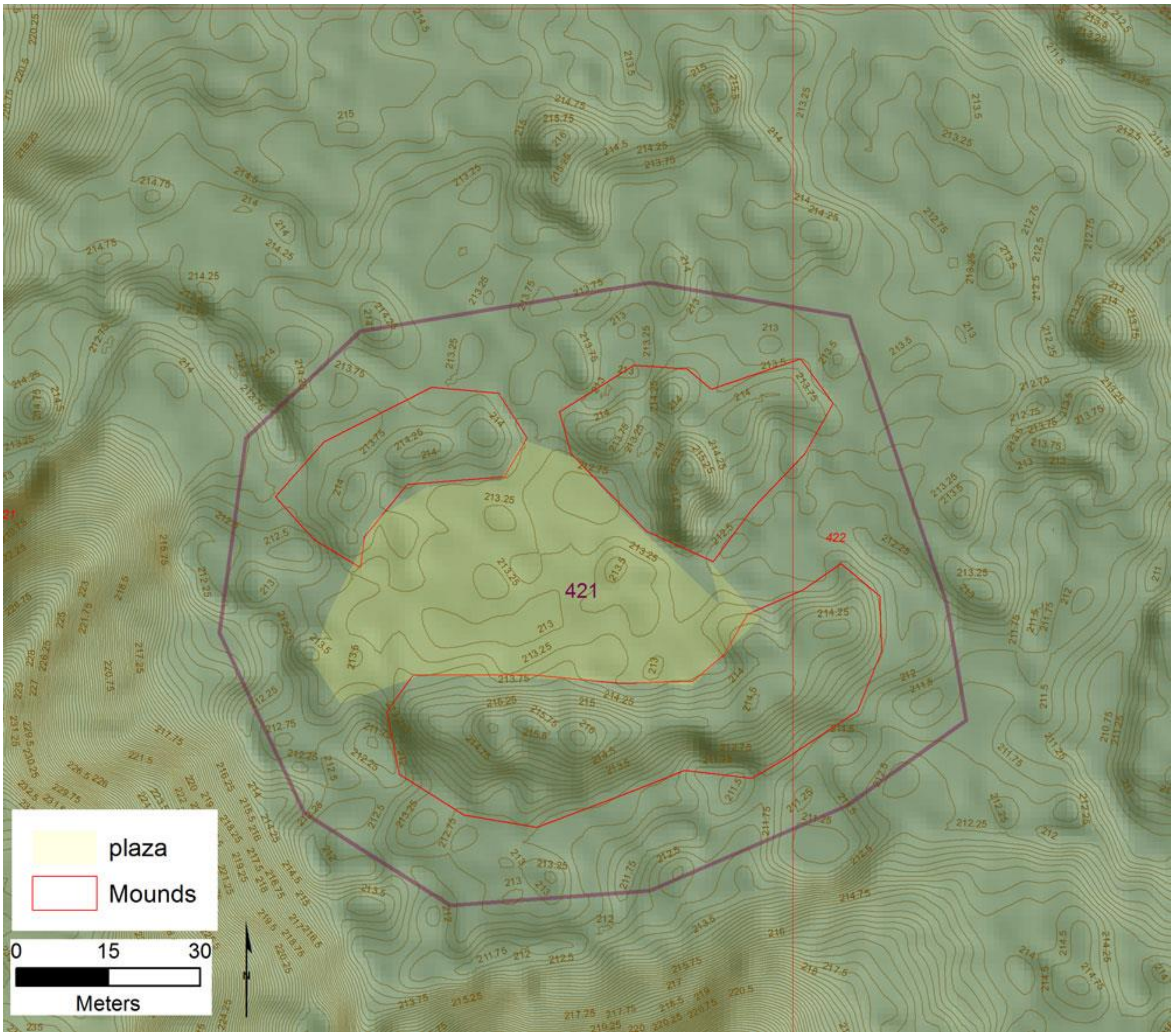

Site 421

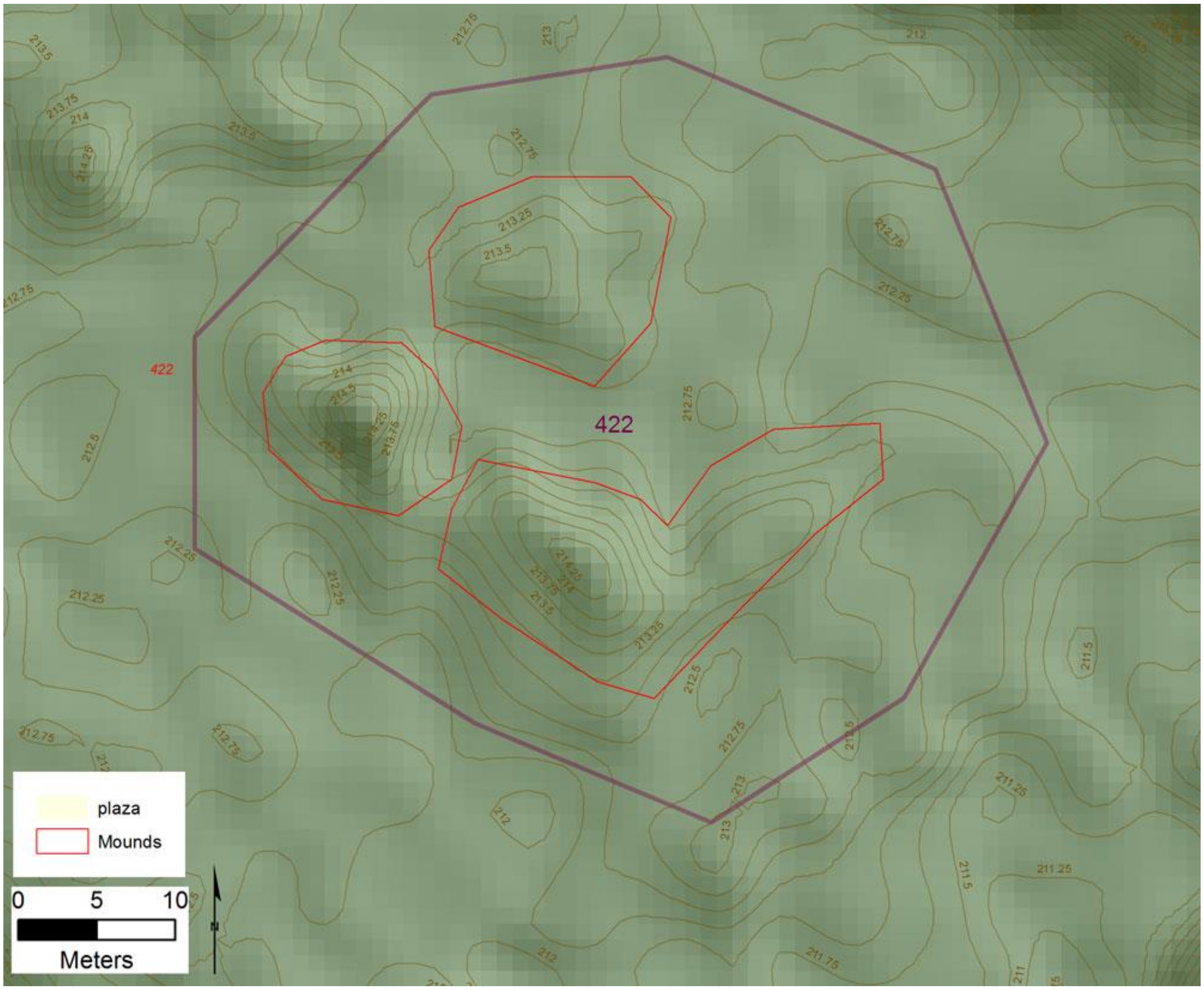

Site 422

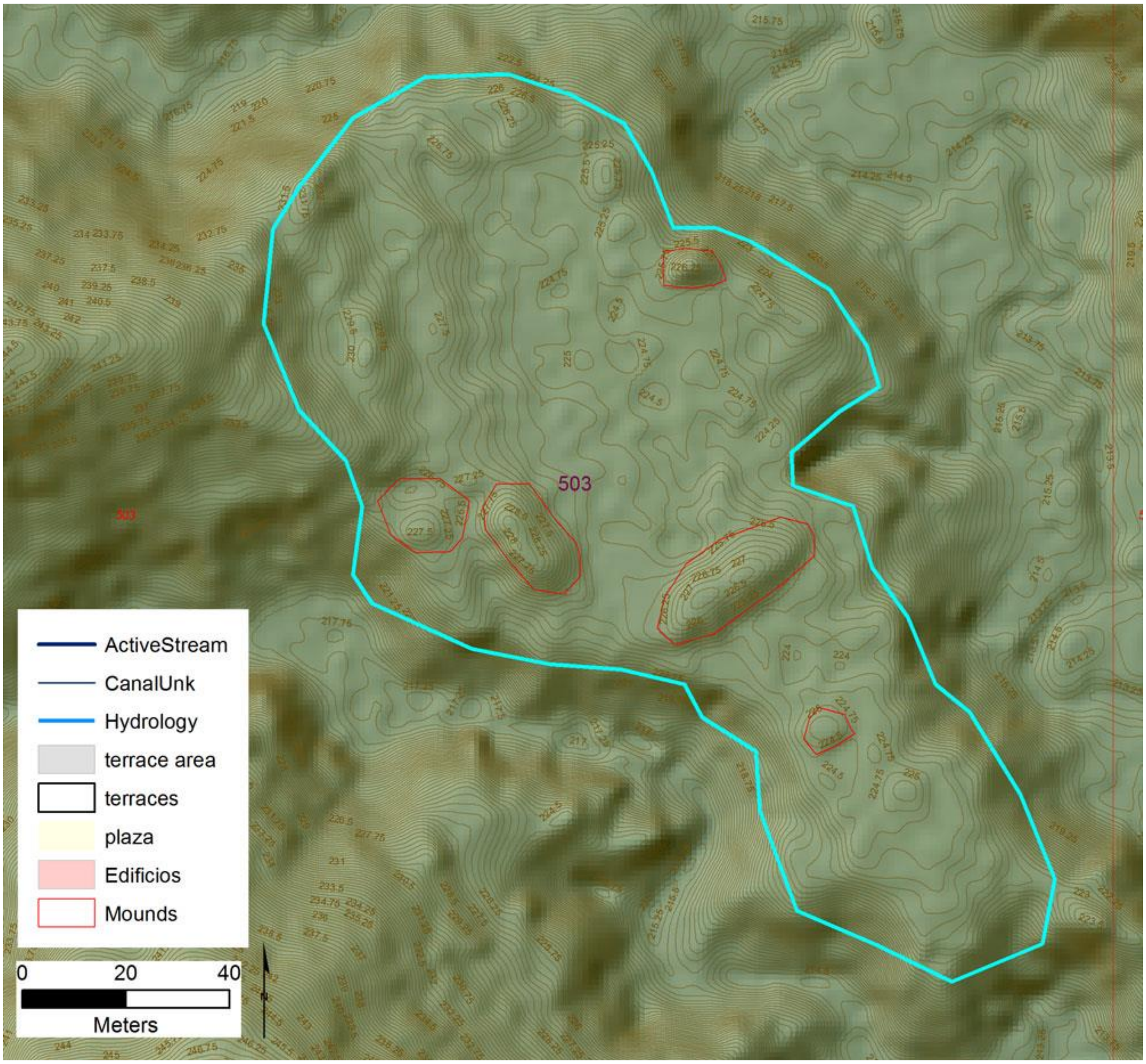
503
ActiveStream
CanalUnk
Hydrology
terrace area
terraces
plaza
Edificios
Mounds
0
20
40
Meters

Site 503

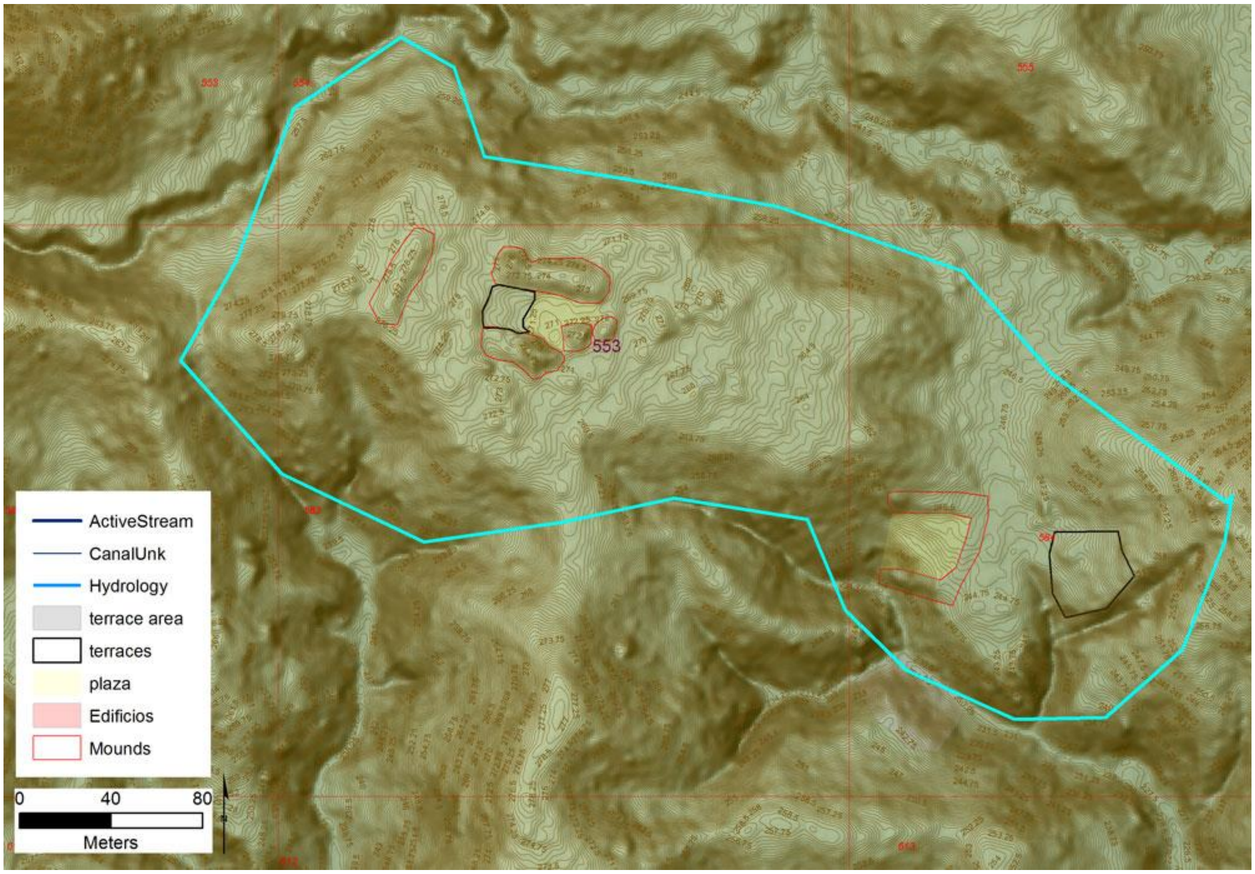
ActiveStream
CanalUnk
Hydrology
terrace area
terraces
plaza
Edificios
Mounds
0
40
80
Meters
553

Site 553

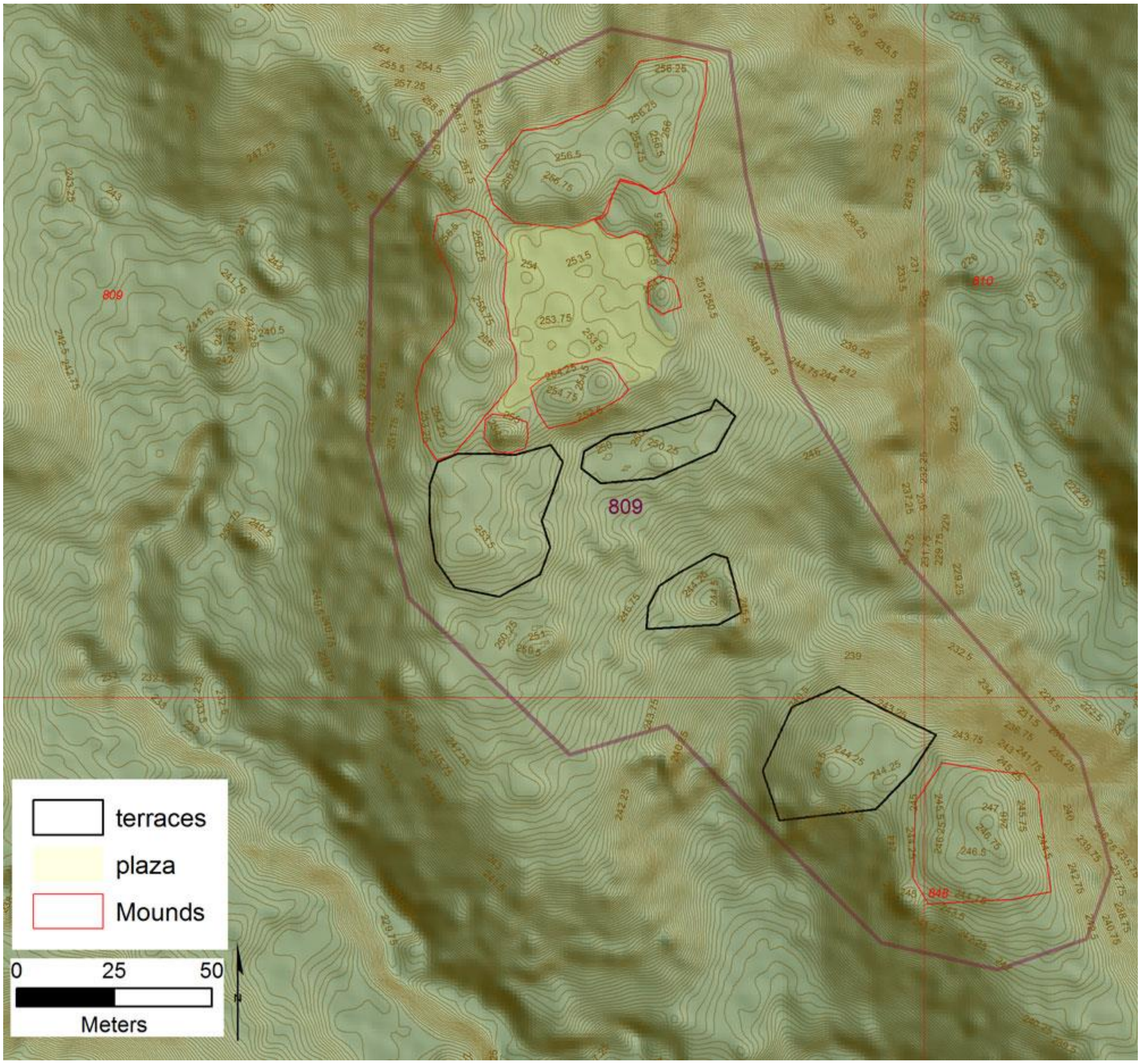

Site 809

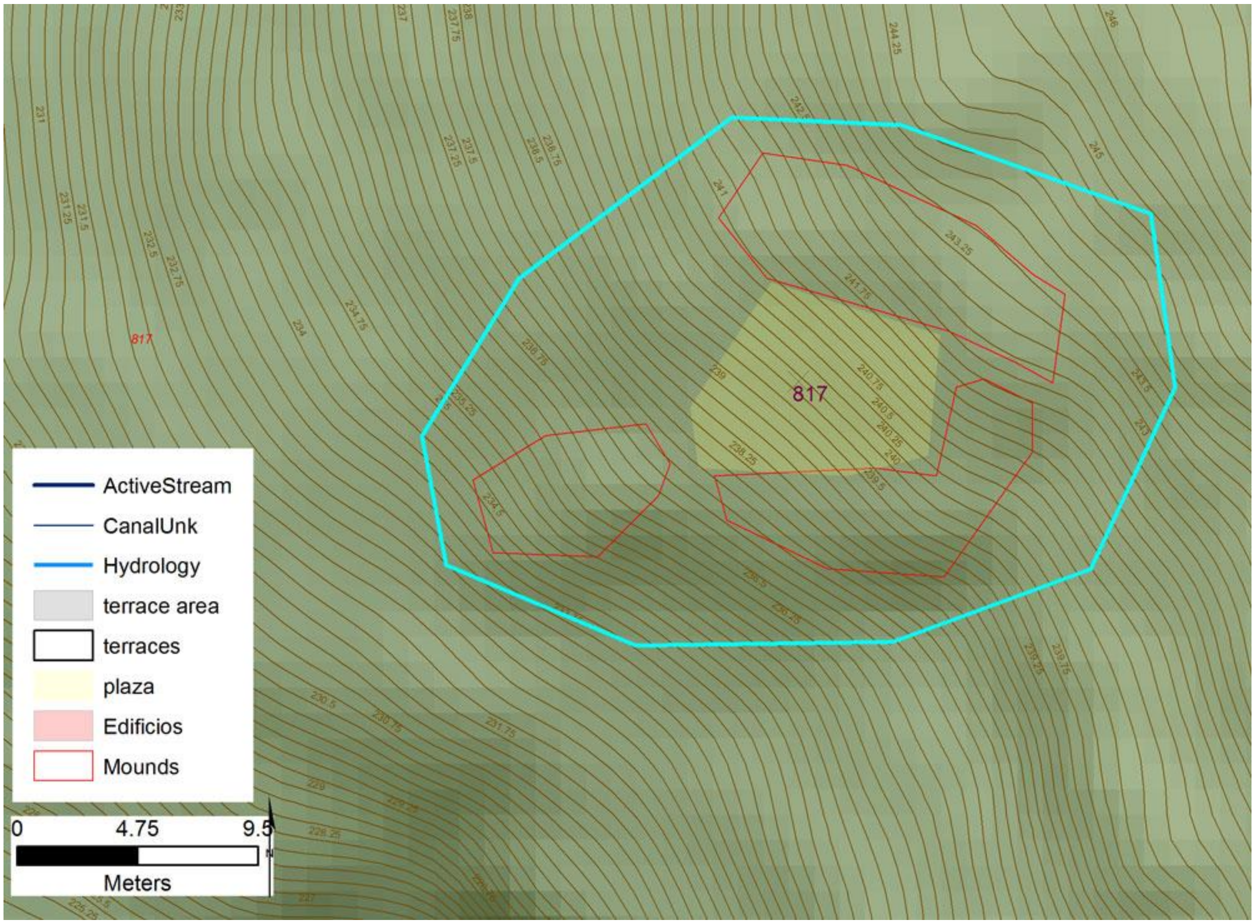
ActiveStream
CanalUnk
Hydrology
terrace area
terraces
plaza
Edificios
Mounds
0
4.75
9.5
Meters
817

Site 817

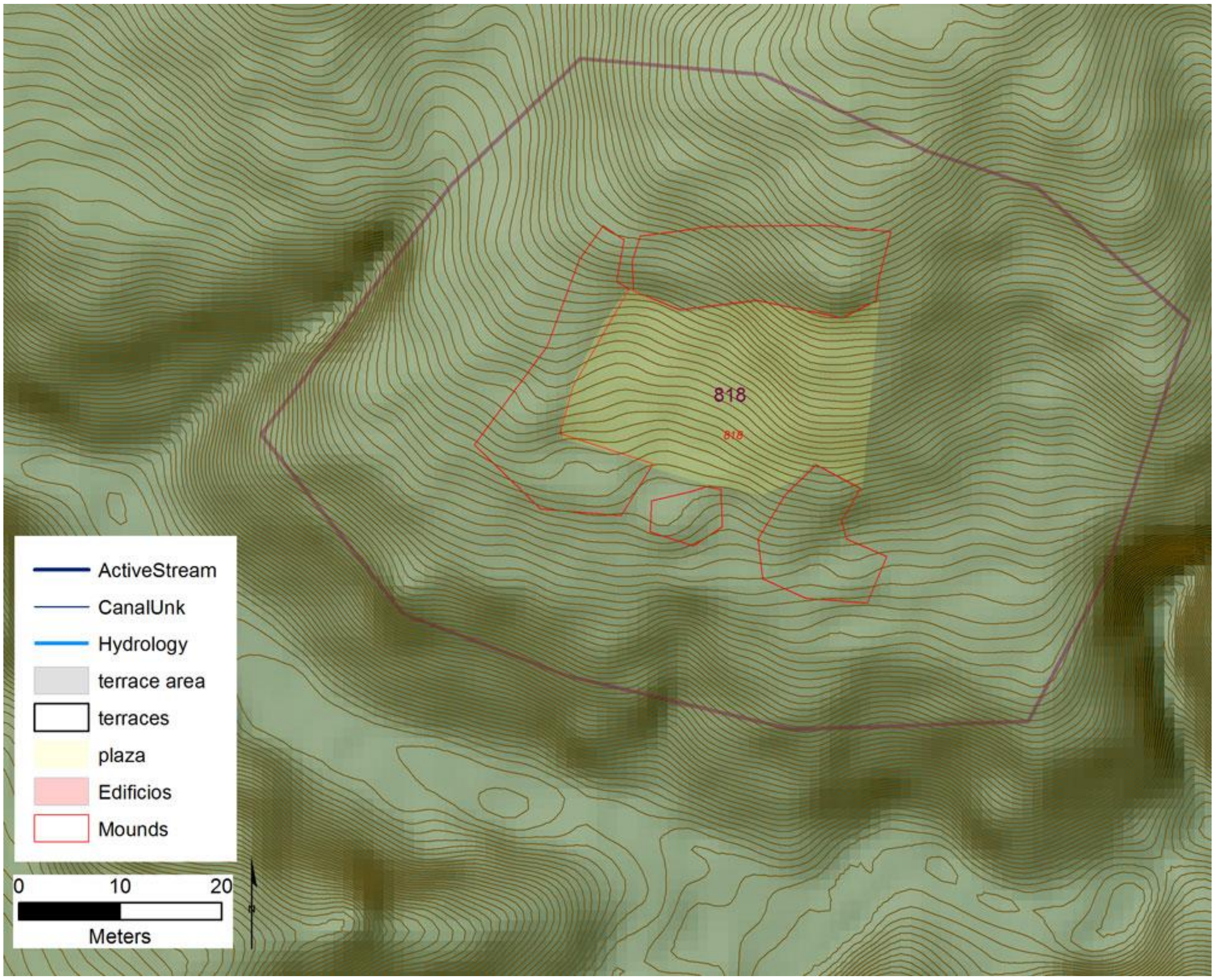
818
ActiveStream
CanalUnk
Hydrology
terrace area
terraces
plaza
Edificios
Mounds
0
10
20
Meters

Site 818

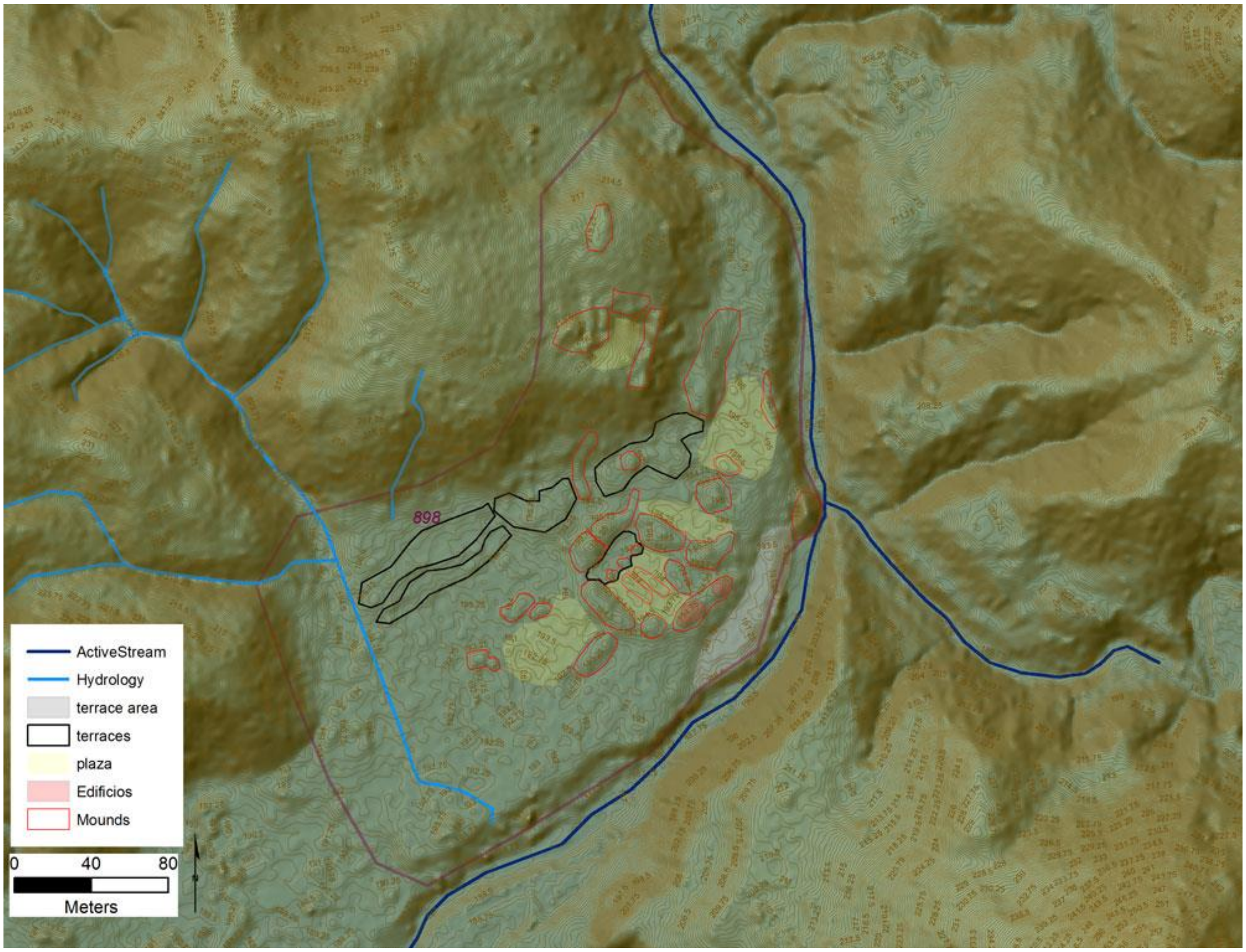

898
ActiveStream
Hydrology
terrace area
terraces
plaza
Edificios
Mounds
0
40
80
Meters

Site 898.

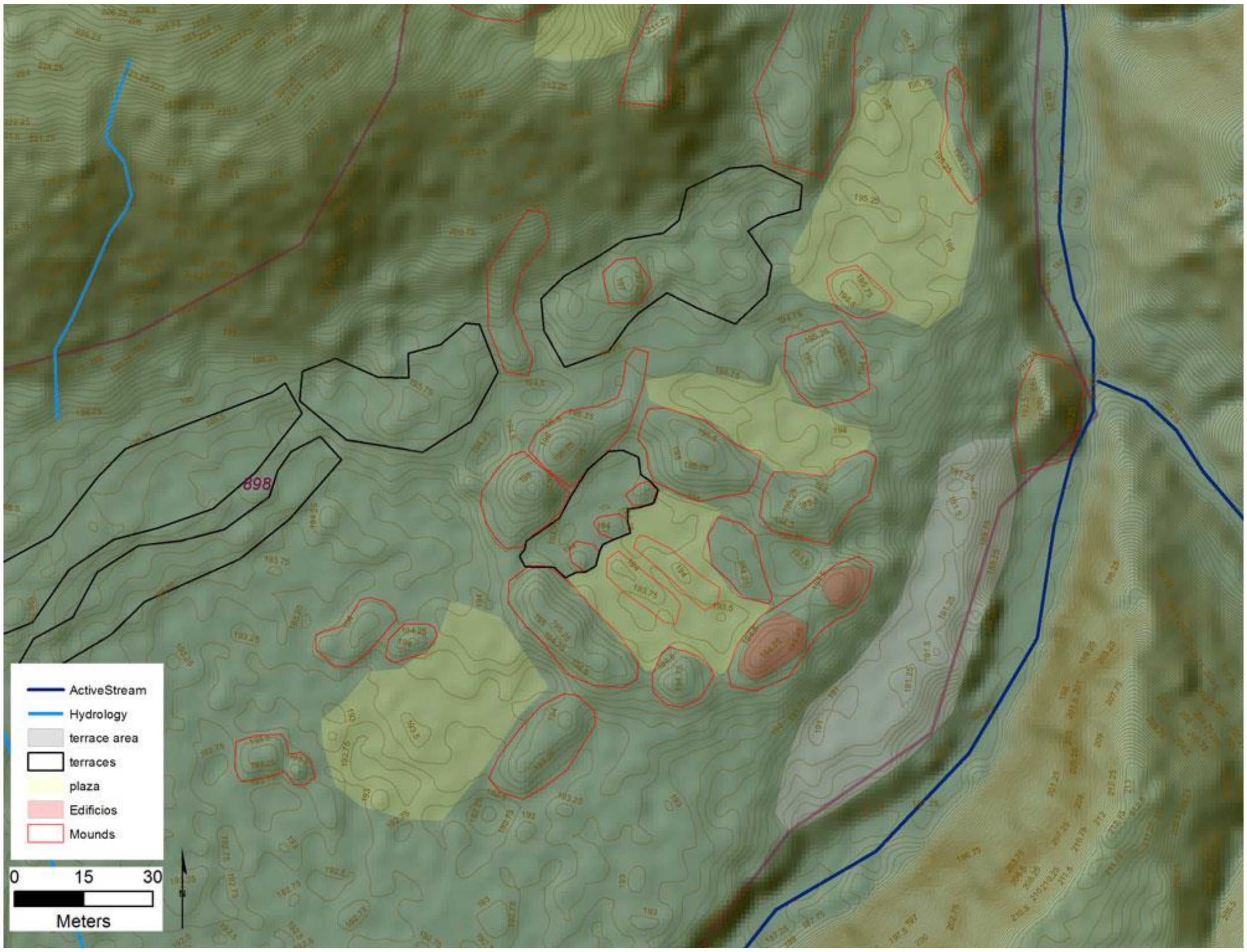

Central Portion of 898.

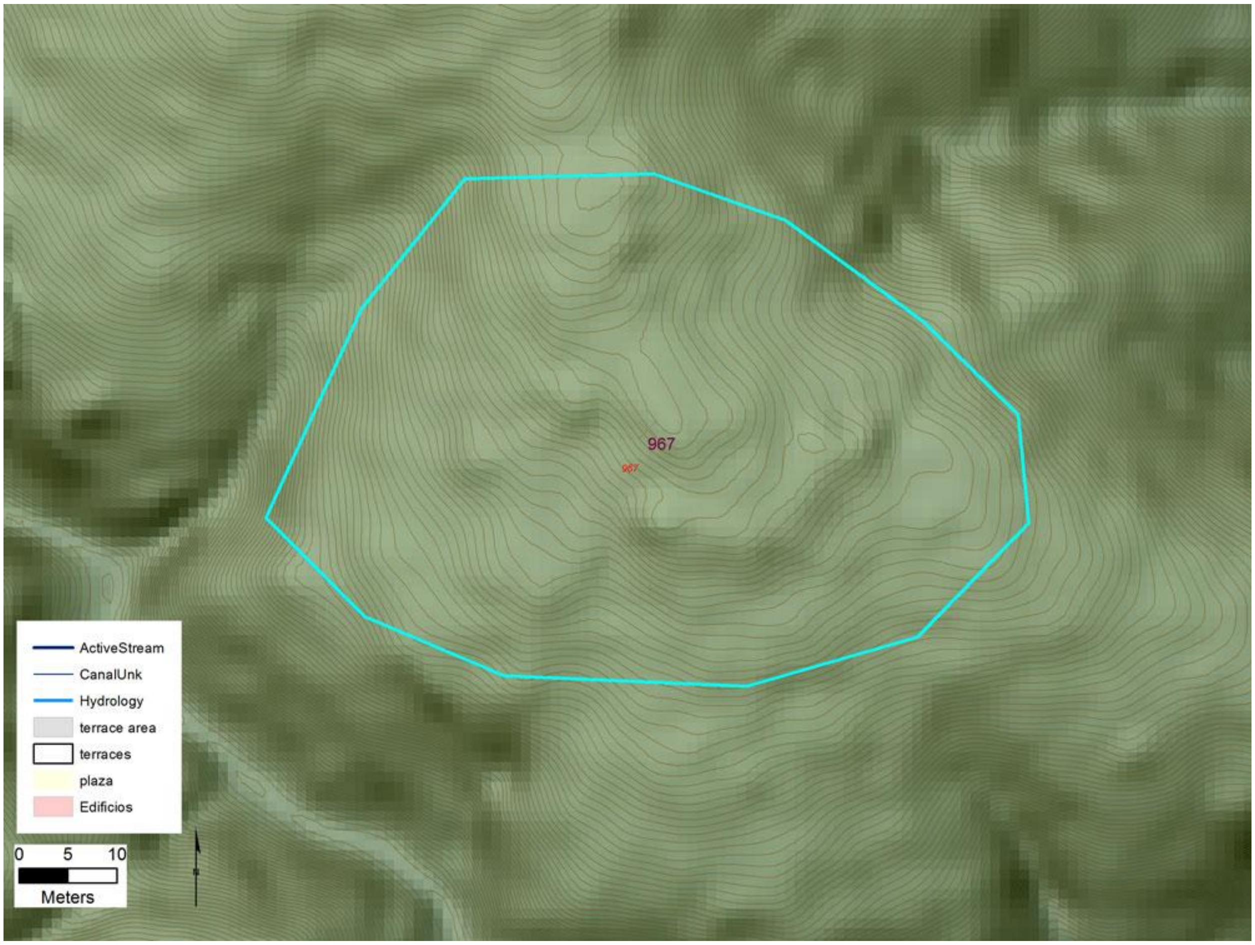
967
ActiveStream
CanalUnk
Hydrology
terrace area
terraces
plaza
Edificios
0 5 10
Meters

Site 967

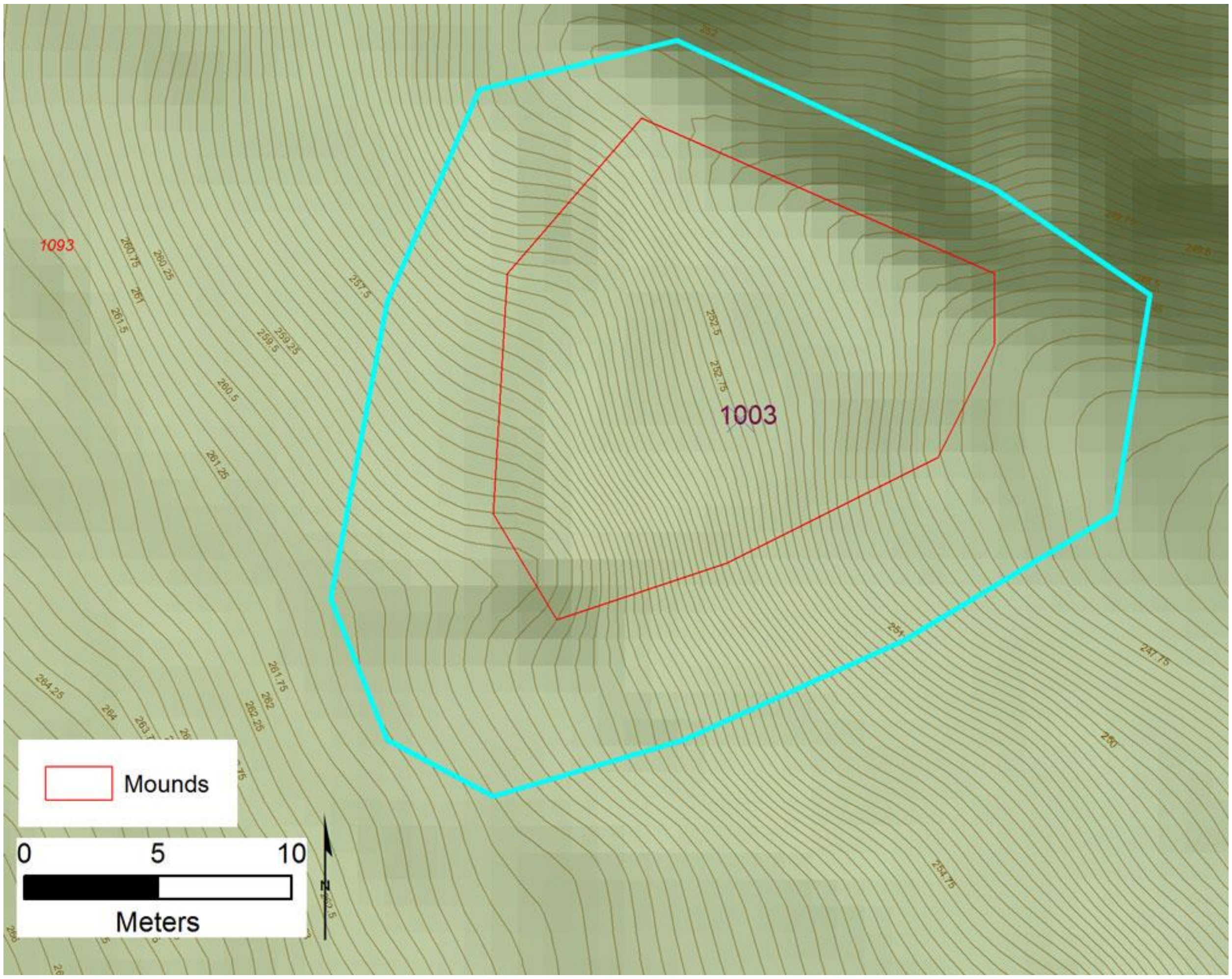

Site 1003

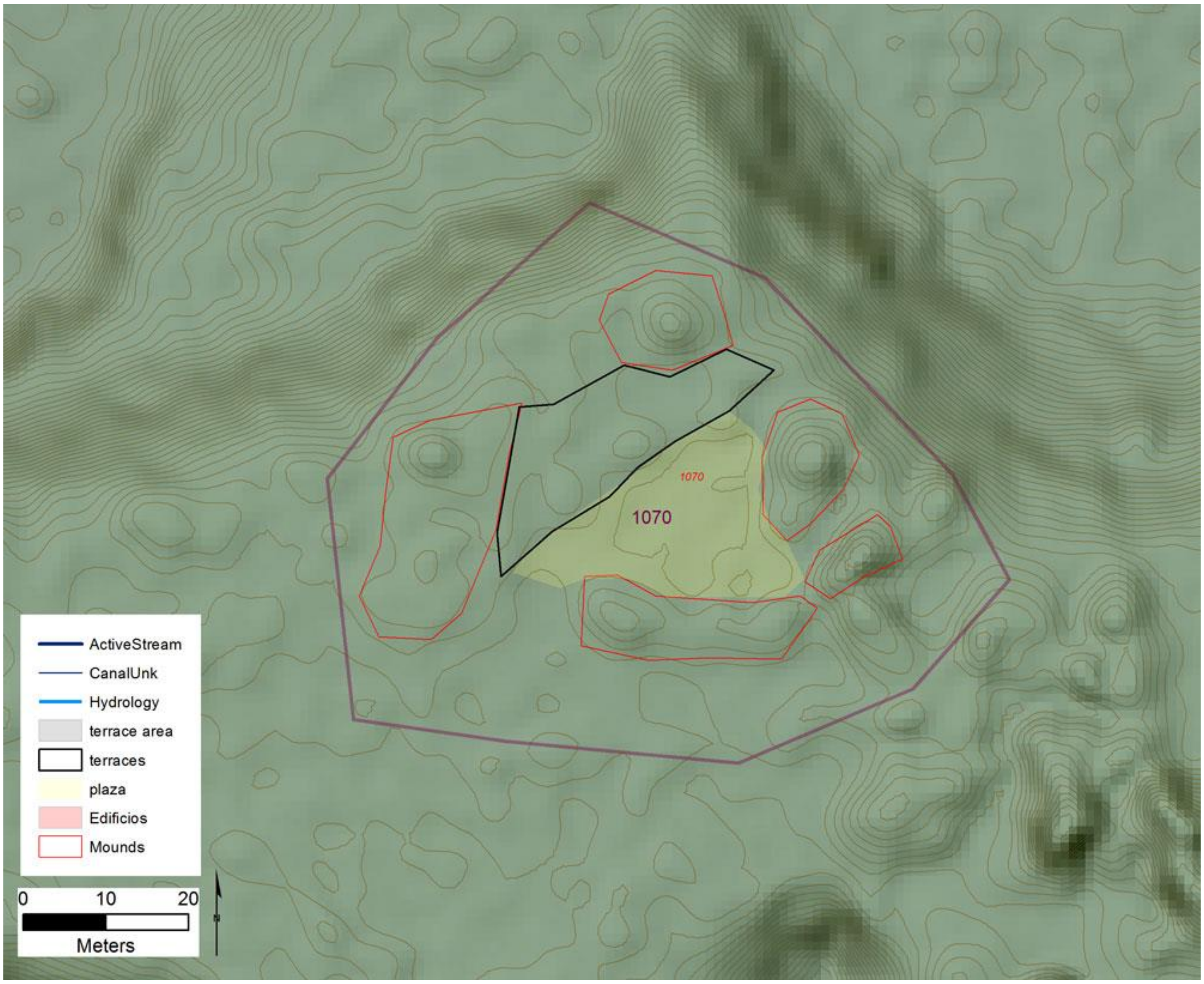
1070
1070
ActiveStream
CanalUnk
Hydrology
terrace area
terraces
plaza
Edificios
Mounds
0
10
20
Meters

Site 1070

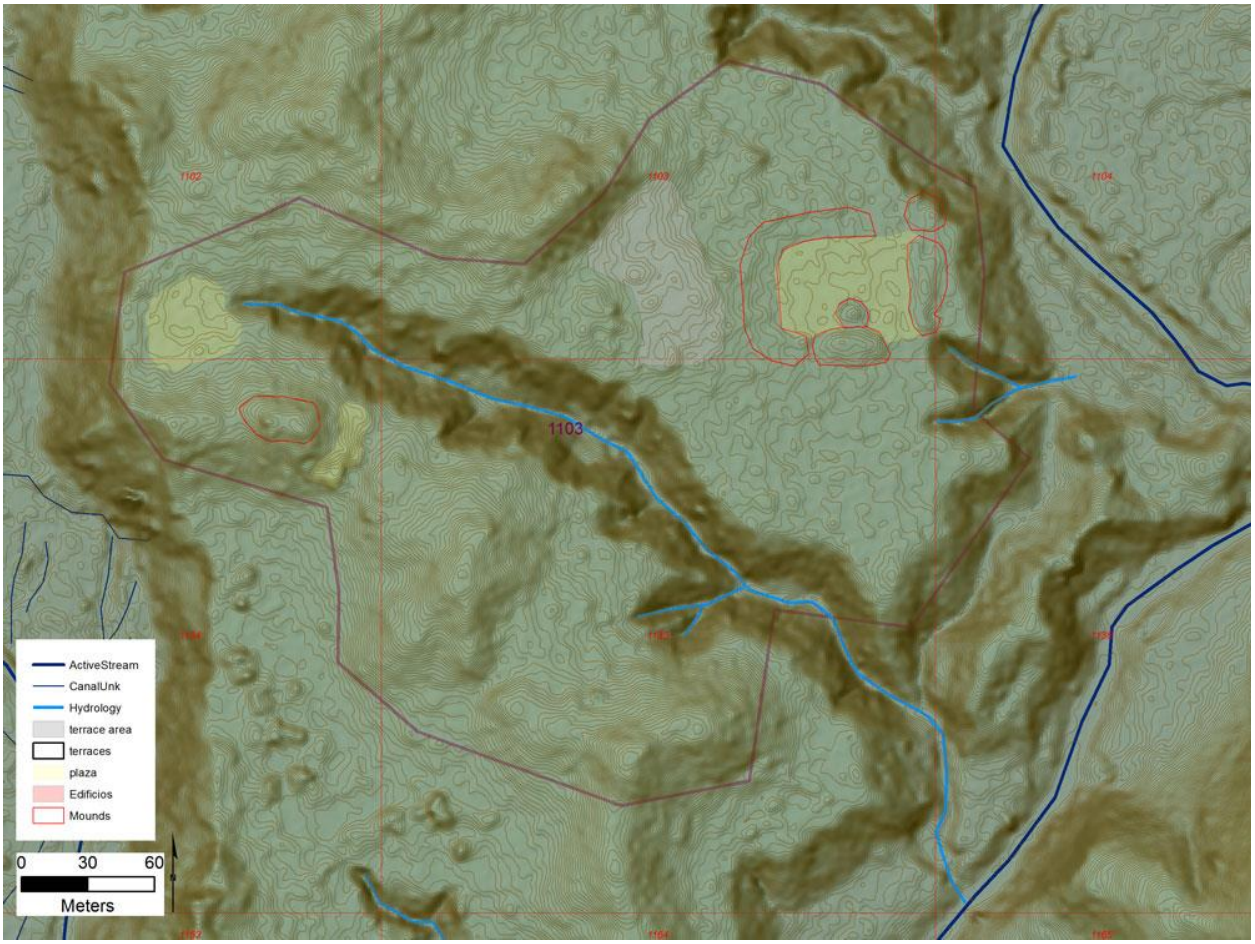
1102
1103
1104
1103
ActiveStream
CanalUnk
Hydrology
terrace area
terraces
plaza
Edificios
Mounds
0
30
60
Meters

Site 1103

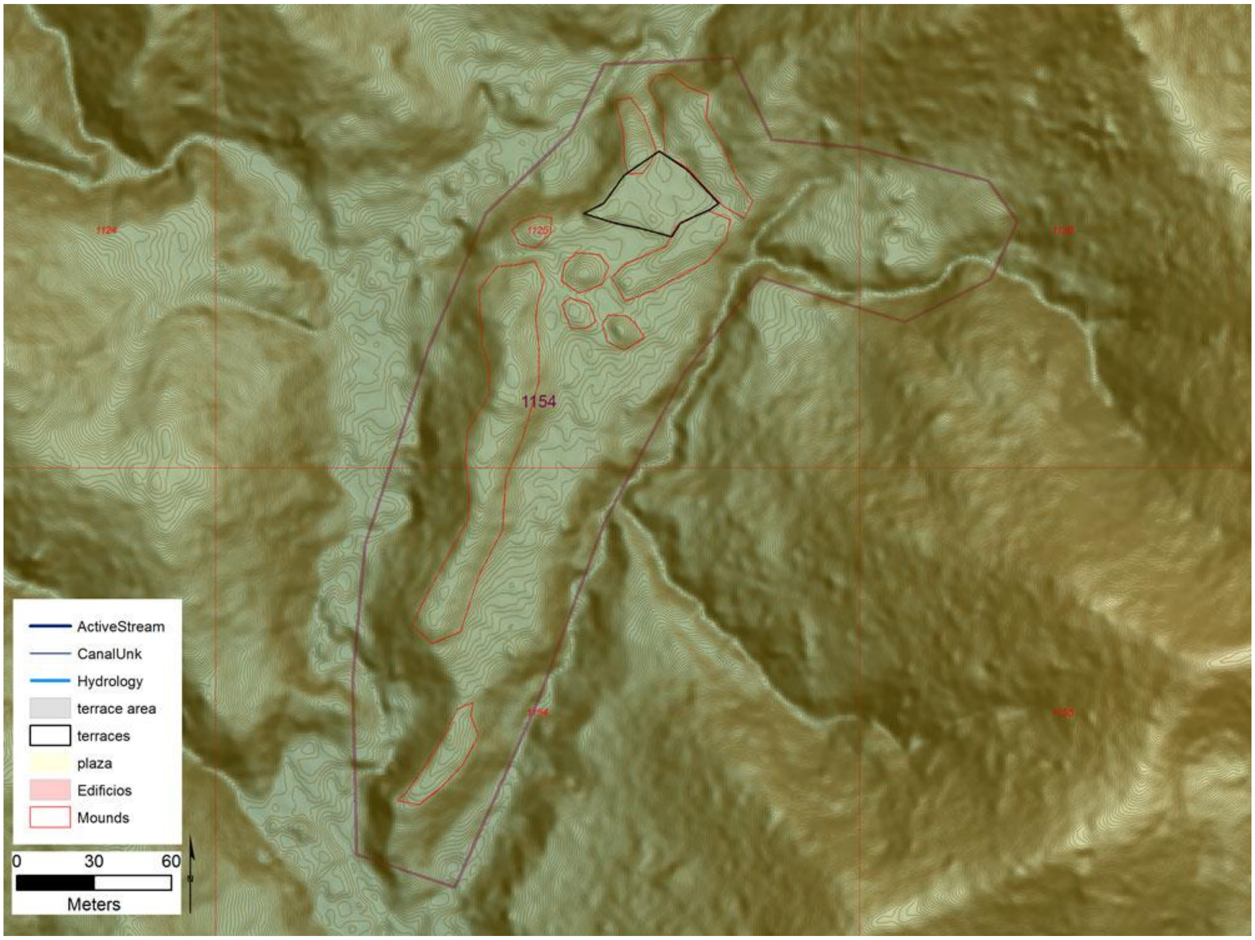
1124
1125
1154
ActiveStream
CanalUnk
Hydrology
terrace area
terraces
plaza
Edificios
Mounds
0
30
60
Meters

Site 1154

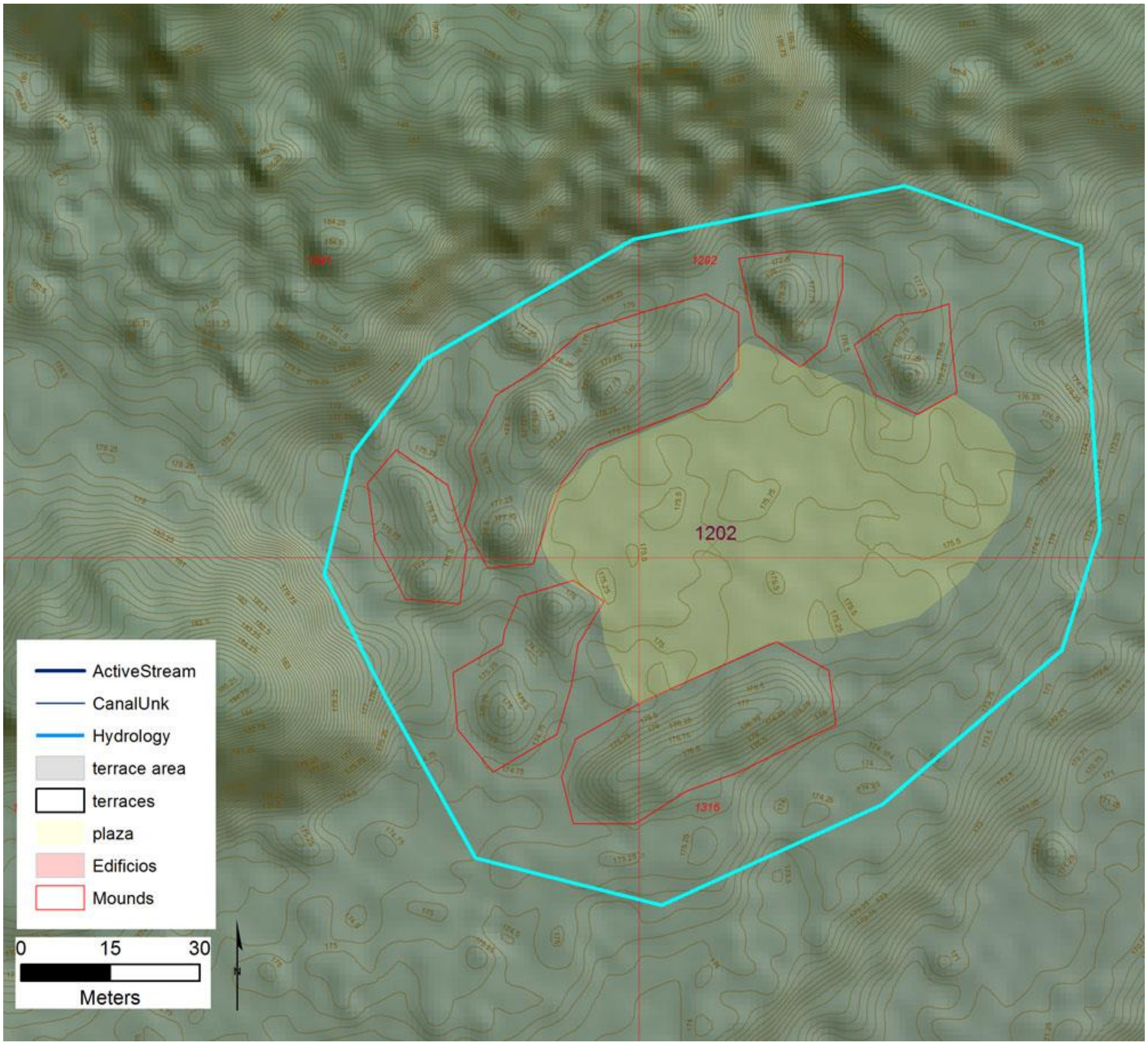
1202
1316
ActiveStream
CanalUnk
Hydrology
terrace area
terraces
plaza
Edificios
Mounds
0
15
30
Meters

Site 1202

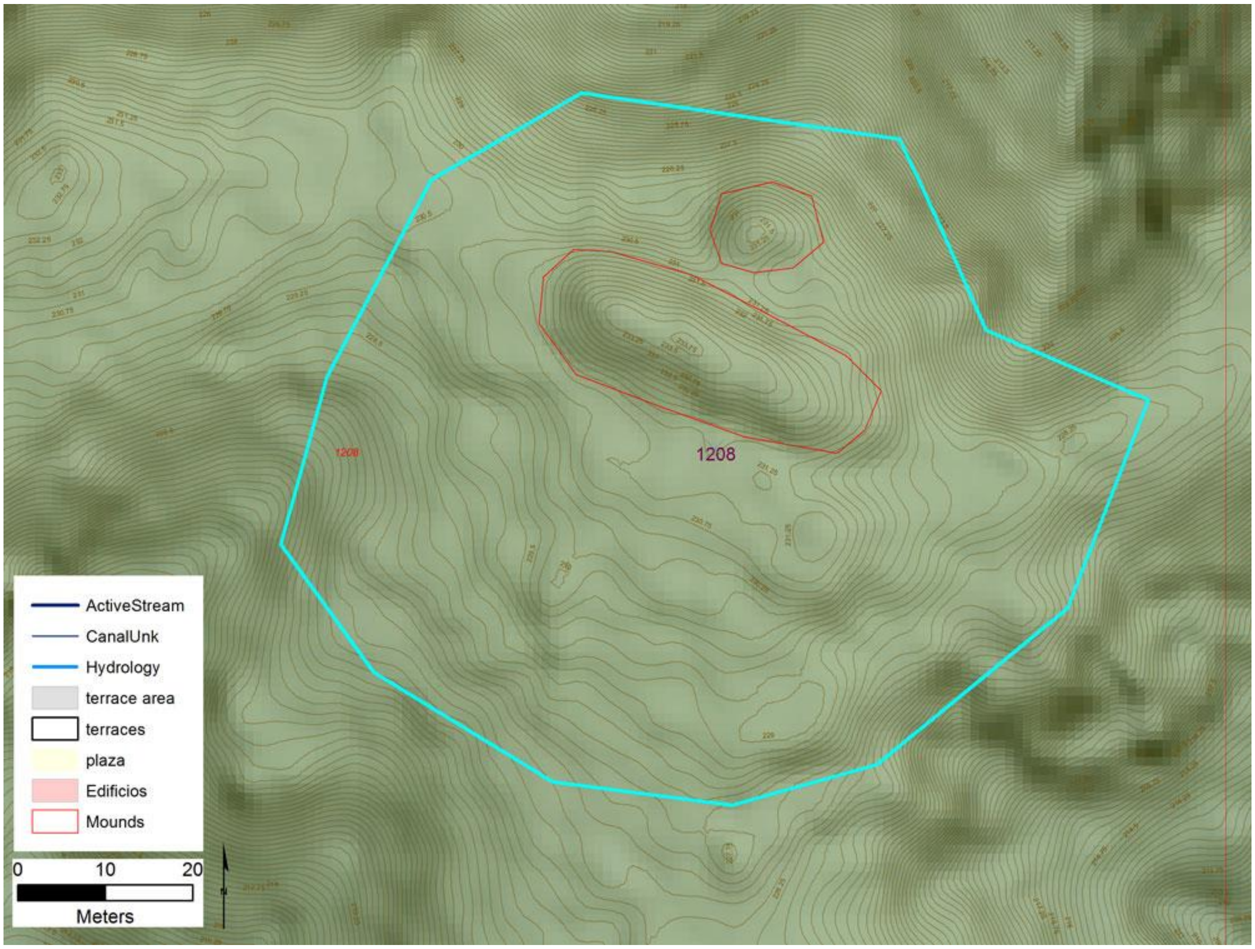
1208
ActiveStream
CanalUnk
Hydrology
terrace area
terraces
plaza
Edificios
Mounds
0
10
20
Meters

Site 1208
